# Tailoring the Surface Integrity of Ti–6Al–4V Alloy by Ultrasonic Surface Rolling Process: A Review

**DOI:** 10.3390/ma19173738

**Published:** 2026-09-02

**Authors:** Guo Li, Xuefei Liu, Siyuan Liu, Hao Chen, Weidong Xie, Guobing Wei

**Affiliations:** 1 International Joint Laboratory for Light Alloys (Ministry of Education), Chongqing University, Chongqing 400044, China; 2 School of Industrial Engineering, Ningxia Vocational and Technical University, Yinchuan 750021, China; 3 College of Materials Science and Engineering, Chongqing University, Chongqing 400044, China; 4 Department of Materials Science and Engineering, University of Toronto, 184 College Street, Toronto, ON M5S 3E4, Canada

**Keywords:** Ti–6Al–4V alloy, ultrasonic surface rolling process, gradient nanostructure, surface integrity, residual compressive stress, fatigue performance

## Abstract

Ti–6Al–4V alloy is widely used in aerospace and other high-performance engineering components, but its service reliability is often constrained by surface-initiated fatigue, fretting damage, wear, and corrosion. Ultrasonic surface rolling process (USRP) couples a static rolling force with high-frequency mechanical impacts to introduce severe plastic deformation while retaining relatively low surface roughness, thereby producing a gradient-strengthened surface layer. This review systematically summarizes advances in USRP strengthening of Ti–6Al–4V alloy within a “process–microstructure–surface integrity–service performance” framework. The effects of static load, ultrasonic amplitude and frequency, feed rate, spindle speed, processing passes, treatment temperature, and lubrication conditions are first compared. Particular attention is then paid to the mechanisms governing dislocation multiplication and rearrangement, grain subdivision, gradient nanostructure formation, the responses of the α and β phases, deformation-induced phase transformation, and the evolution of depth-dependent residual compressive stress. The intrinsic relationships between these mechanisms and surface roughness, hardness, strengthened layer depth, wear and corrosion resistance, fatigue performance, and fretting fatigue performance are subsequently clarified. Control strategies involving electropulsing, laser/temperature assistance, deep cryogenic treatment, and coating combinations are further reviewed, together with methods for contact dynamics analysis, residual stress prediction, and data-driven optimization. The combined evidence indicates that the performance gains from USRP are jointly controlled by surface defects, gradient microstructure, and residual compressive stress; excessive load, processing passes, or heat input may weaken or even reverse the fatigue benefit because of defect accumulation, gradient mismatch, and residual stress relaxation. Current limitations include inconsistent reporting of process parameters, difficulty in quantitatively separating the contributions of different strengthening mechanisms, insufficient investigation of residual stress stability, and limited validation on complex components.

## 1. Introduction

Ti–6Al–4V is a typical α + β dual-phase titanium alloy. Owing to its high specific strength, good corrosion resistance, excellent damage tolerance, and broad service temperature range, it is widely used in aero-engine blades, compressor disks, blisks, aircraft landing gear, fasteners, and other high-specific-strength load-bearing components [1,2]. Nevertheless, Ti–6Al–4V has relatively limited surface hardness and wear resistance and exhibits high notch sensitivity. Under the combined action of cyclic loading, contact loading, and corrosive media, components are susceptible to surface wear, fretting damage, stress corrosion, and fatigue cracking [3,4,5]. Because fatigue cracks usually initiate preferentially at surface machining defects, contact-damaged regions, or local stress concentrations, the condition of the surface layer often governs fatigue life and service reliability more directly than the static strength of the bulk material [3,4]. Controlling the surface condition of Ti–6Al–4V without significantly changing the overall component dimensions or bulk microstructure is, therefore, an important scientific and engineering route to improved long-term performance.

Surface integrity is not a single surface roughness metric but a multiscale state comprising surface morphology, microscopic defects, work hardening, gradient microstructure, residual stress, and their depth distributions [3,6,7]. These factors act competitively and synergistically during crack initiation and propagation. Lower surface roughness reduces geometric stress concentration; higher surface hardness suppresses local plastic deformation and abrasive ploughing; a gradient-refined microstructure strengthens the surface and modifies the crack path; and residual compressive stress of sufficient magnitude, depth, and stability reduces the effective tensile stress and crack tip driving force under cyclic loading. Conversely, surface folding, microcracking, spalling, an excessively steep hardness gradient, or unstable residual stress caused by overprocessing may offset the benefits of microstructural strengthening. The effectiveness of surface strengthening in Ti–6Al–4V, therefore, cannot be assessed from the maximum value of any single parameter. Instead, an integrated surface integrity framework linking surface morphology, gradient microstructure, mechanical state, and service performance is required.

Shot peening, laser shock peening, deep rolling, surface mechanical attrition, and ultrasonic impact treatments have been used to improve the surface integrity of titanium alloys. Shot peening is relatively mature in engineering practice and can introduce work hardening and residual compressive stress, but random shot impacts may also increase surface roughness and generate folds, dimples, or local microdamage. A comparative study on Ti–6Al–4V showed that both shot peening and ultrasonic surface rolling improved fretting fatigue performance, whereas ultrasonic surface rolling produced a smoother surface, a deeper residual compressive stress layer, and more pronounced surface grain refinement [4]. Thus, an ideal surface-strengthening process must not only impart sufficient plastic deformation but also control surface material flow, defect formation, and stress gradients to integrate surface smoothing with subsurface strengthening.

Ultrasonic surface rolling process (USRP) couples a static rolling force, high-frequency ultrasonic vibration, and rolling feed motion. Periodic high-frequency contact between the rolling head and workpiece produces repeated indentation, plastic flow, and filling of surface valleys. Compared with purely static rolling, ultrasonic vibration increases the instantaneous impact energy and surface plastic strain rate and promotes the transmission of plastic deformation into the material. Static–dynamic load separation experiments and numerical simulations showed that single-point cyclic dynamic indentation can produce nominal strain rates on the order of 10^3^ s^−1^ in the surface layer of Ti–6Al–4V, and that the peak contact force during USRP is not a simple linear sum of the static load and maximum dynamic load [8]. This result was obtained under simplified single-point impact conditions and should not be regarded as a universal strain rate for all continuous rolling conditions. Contact mechanics and finite element studies further showed that static load, rolling head dimensions, amplitude, rolling trajectory, and repeated coverage jointly determine the plastic zone size and residual stress distribution [9]. The strengthening effect of USRP is, therefore, governed by the coupling among contact load, energy input per unit area, strain rate, trajectory coverage, and repeated deformation, rather than by any single machine parameter.

The severe plastic deformation introduced by USRP can form a gradient structure in the surface layer of Ti–6Al–4V consisting of nanograins, nanolamellae, ultrafine grains, and deformed coarse grains, thereby combining high surface strength with deformation compatibility in the interior [10,11]. Because Ti–6Al–4V contains hexagonal close-packed α and body-centered cubic β phases, the two phases differ markedly in slip systems, critical resolved shear stress, and plastic deformability, and consequently follow different grain refinement paths. Gradient refinement of the α phase mainly proceeds through dislocation multiplication, dislocation wall formation, subgrain boundary evolution, lamellar subdivision, and dynamic recovery [11]. The β phase is progressively refined into nanoscale grains primarily through dislocation activity and does not necessarily require twinning [12]. Some studies have also observed an hcp → fcc titanium transformation induced by severe surface plastic deformation and proposed that interactions among phase transformation, twinning, and dislocations may contribute to local refinement [13]. However, fcc-Ti formation depends on the initial microstructure, local strain, strain rate, and thermodynamic conditions and should not be generalized as a universal mechanism under all USRP conditions. Microstructural evolution in USRP-treated Ti–6Al–4V must, therefore, be discussed within a framework of coordinated α/β deformation and depth-dependent strain partitioning.

Gradient microstructure, work hardening, improved surface morphology, and residual compressive stress collectively constitute the principal physical basis for the fatigue improvement produced by USRP [3,14], although their relative contributions remain debated. Some studies consider deep residual compressive stress to be the dominant factor in retarding fatigue crack propagation because it reduces the effective stress intensity factor at the crack tip [14,15]. Others indicate that, after cyclic relaxation of residual stress, gradient grains, dislocation substructures, and phase interfaces can still disperse local plastic strain, increase crack growth resistance, and increase crack path tortuosity [16]. Fretting fatigue studies further suggest that residual compressive stress contributes more strongly to crack propagation life than to crack initiation life, whereas surface roughness, hardness, and the fretting wear state remain important to crack initiation [15]. Fatigue enhancement, therefore, cannot be attributed simply to either “grain refinement” or “increased residual compressive stress”; crack initiation and propagation must be distinguished and analyzed quantitatively in conjunction with residual stress stability and microstructural evolution.

Existing studies also show that the relationships between USRP parameters, surface integrity, and fatigue performance are generally non-monotonic. Spindle speed, static load, amplitude, feed rate, rolling passes, and coverage all affect surface material flow, plastic deformation depth, and residual stress state [7,17,18,19]. For example, an appropriate parameter combination reduced the surface roughness Ra of TC4 alloy to 0.052 μm, produced an axial surface residual compressive stress of approximately −685 MPa, and increased fatigue strength from 280 to 425 MPa. However, fatigue lives varied markedly among parameter combinations, demonstrating that increases in surface hardness or residual compressive stress do not necessarily produce continuous improvements in fatigue life [7]. Multipass studies likewise found that an intermediate number of rolling passes provides a favorable balance of fatigue and corrosion performance, whereas further passes do not guarantee additional improvement [18]. Recent work has further linked amplitude to impact kinetic energy, high-strain-rate plastic deformation, low-angle grain-boundary evolution, and fatigue life, indicating the need to progress from empirical single-factor comparisons to a unified description based on energy input and deformation state [19].

As research has progressed, USRP has expanded from conventionally forged or rolled Ti–6Al–4V specimens to additively manufactured components, welded joints, coating substrates, and complex curved parts. For selective electron beam-melted Ti–6Al–4V, USRP can flatten partially melted particles and melt pool ridges, reduce surface roughness, and form a relatively deep hardened layer [20]. For Ti–6Al–4V laser-welded joints, ultrasonic rolling improves weld surface morphology, regulates welding residual stress, and reduces the fatigue crack growth rate [21]. USRP has also been used as a pretreatment for plasma electrolytic oxidation to mitigate the adverse effects of coating defects and residual tensile stress on fatigue performance [22]. Electropulsing-assisted USRP can exploit electroplasticity to enlarge the effective plastic deformation zone, although an appropriate window must be maintained between heat input and strengthening [23]. Combining low-plasticity ultrasonic rolling with five-axis numerical control machining has shown potential for improving the surface morphology and residual stress uniformity of near-net-shaped blades [24]. These advances indicate that USRP is evolving from a surface-strengthening method for regular specimens into a surface integrity manufacturing technique for complex components.

Although USRP has been demonstrated to regulate the surface integrity of Ti–6Al–4V effectively, several important limitations remain. Most importantly, a quantitatively validated and transferable relationship has not yet been established between machine-level USRP parameters, the effective local deformation state, the coupled depth-dependent evolution of surface integrity, and the resulting service performance. Consequently, it remains difficult to predict from a given parameter combination whether the treatment will produce a favorable balance among surface morphology, α/β microstructure, hardness, and residual compressive stress, or to define a robust processing window that distinguishes insufficient treatment, effective strengthening, and overprocessing. This central gap is reinforced by the inconsistent use of the terms USRP, URSP, SUR, UNSM, and ultrasonic rolling strengthening, even though the corresponding equipment configurations, loading modes, and energy inputs are not necessarily equivalent. The incomplete reporting of static load, amplitude, frequency, rolling head diameter, feed rate, trajectory spacing, coverage, processing passes, lubrication conditions, and temperature rise further hinders quantitative comparison among studies. Moreover, the individual contributions of surface roughness, gradient microstructure, work hardening, and residual compressive stress to fatigue, fretting, wear, and corrosion performance have not been reliably separated, while the stability of residual compressive stress under cyclic loading, elevated temperature, and corrosive environments remains insufficiently understood. Most available experiments also involve simple shafts or flat specimens, leaving dimensional effects, processing uniformity, deformation control, and quality evaluation criteria for thin-walled blades, welded joints, additively manufactured components, and complex surfaces inadequately validated. Taken together, these limitations indicate that the principal need is not merely a broader inventory of reported USRP outcomes, but a Ti–6Al–4V-specific synthesis that connects processing conditions with deformation mechanisms, depth-dependent surface integrity, and service performance.

Against this background, the distinction between the present review and the existing review literature becomes clear. Previous reviews of titanium alloy surface engineering have generally emphasized comparisons among multiple surface treatment routes and their overall effects on surface properties [2]. Although such broad comparisons provide valuable context, they do not fully resolve the material-specific questions identified above. Accordingly, the present review focuses specifically on USRP-treated Ti–6Al–4V and organizes the available evidence along the chain of “process parameters–contact and deformation mechanisms–α/β microstructure and phase evolution–depth-dependent surface integrity–service performance.” Particular attention is given to phase-dependent refinement pathways, non-monotonic processing effects, deterioration caused by overprocessing, residual stress formation and stability, and the different roles of surface integrity in fatigue crack initiation and propagation. This approach shifts the emphasis from simply asking whether USRP is beneficial to determining why, when, and through which coupled mechanisms reliable performance improvements can be achieved.

**Figure 1:**
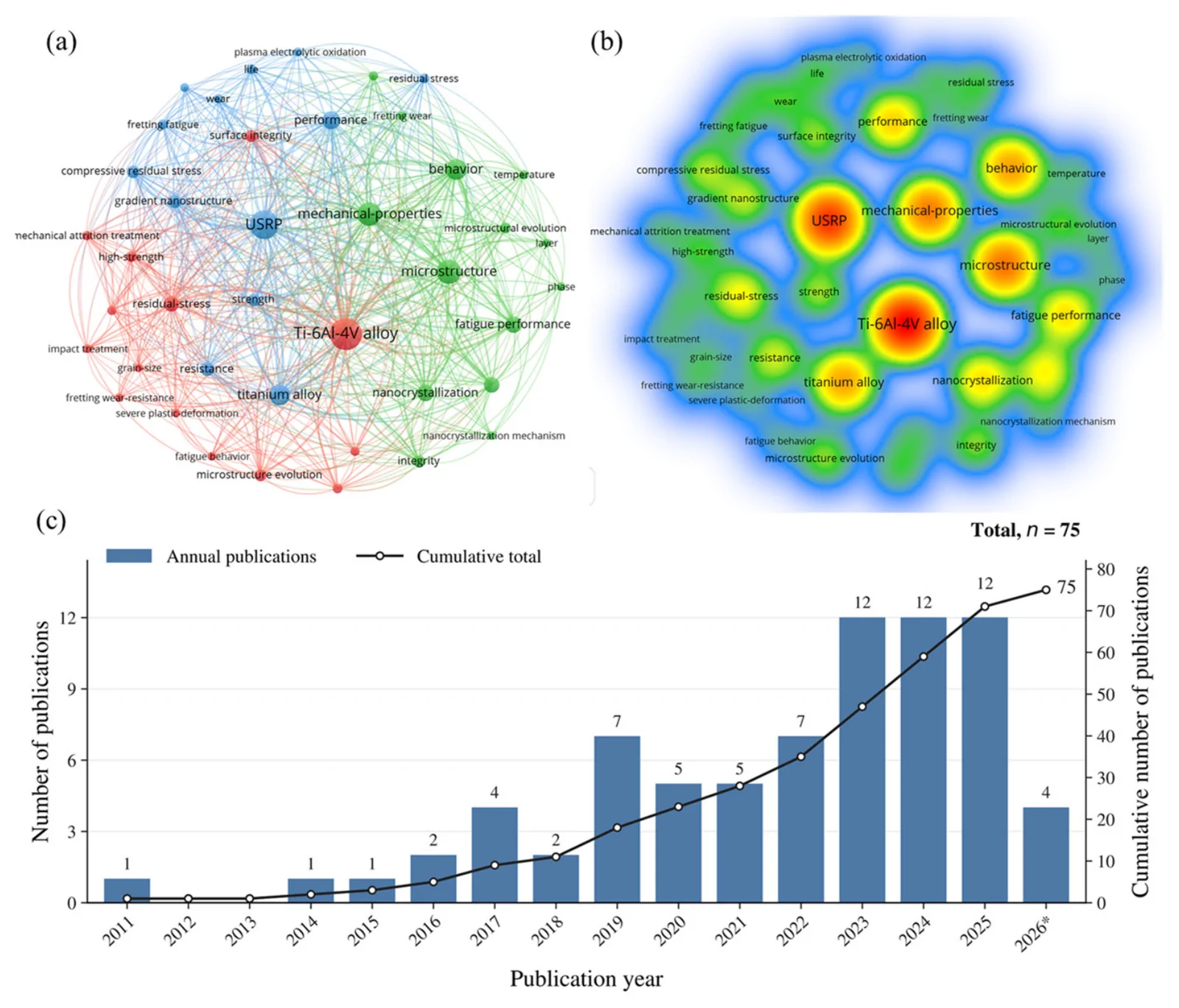
Thematic co-occurrence characteristics of USRP research on Ti–6Al–4V alloy (*n* = 75): (**a**) co-occurrence network of core keywords; (**b**) corresponding keyword density map; and (**c**) annual and cumulative numbers of publications (search updated through July 2026).

To implement this material-specific and mechanism-centered scope, a structured literature search and citation-tracking procedure was applied through July 2026, yielding an evidence set comprising 75 core studies together with mechanistic and background references; its annual distribution and cumulative growth are shown in Figure 1. The overarching objective of this review is to establish an evidence-based understanding of how machine-level USRP parameters and the effective local deformation input govern the coupled evolution of surface morphology, α/β microstructure and phase state, hardness, and residual stress, and how these changes determine the reliability of service performance improvements in Ti–6Al–4V. Specifically, this review aims to: (i) establish a consistent basis for comparing USRP processing parameters and the corresponding contact, energy-input, and deformation states; (ii) clarify the phase-dependent mechanisms governing α/β grain refinement, local phase transformation, work hardening, and depth-dependent residual stress formation; (iii) critically assess the coupled and competing effects of surface morphology, gradient microstructure, hardness, and residual stress on fatigue, fretting, wear, and corrosion performance, including the conditions under which overprocessing may weaken or reverse the expected benefit; and (iv) identify the principal evidence gaps and define research priorities for parameter standardization, residual stress stability, multiscale simulation, physics–data-integrated optimization, and validation on complex components. These objectives determine the organization of the following sections, which progress from process fundamentals and deformation mechanisms to surface integrity evolution, service performance, emerging control strategies, and future research priorities.

## 2. Research Evolution and Hotspot Distribution of USRP for Ti–6Al–4V Alloy

To construct the literature dataset used in Figure 1, a structured topic search was conducted in the Web of Science Core Collection, restricted to the Science Citation Index Expanded (SCI-EXPANDED, 1900–present) and the Social Sciences Citation Index (SSCI, 1900–present). “Ti–6Al–4V alloy” and “Ultrasonic Surface Rolling Process” were used as the core search terms. The search was completed in July 2026 and was limited to English-language journal articles and reviews. Forward and backward citation tracking was additionally performed. After duplicate removal, the retrieved records were sequentially screened by title, abstract, and full text. Studies directly addressing ultrasonic surface rolling of Ti–6Al–4V/TC4 alloy, together with a limited number of publications required for terminology definition, mechanistic interpretation, and engineering comparison, were retained, yielding a final dataset of 75 records. Keyword co-occurrence analysis was performed using VOSviewer 1.6.19. “All keywords” was selected as the unit of analysis, the full-counting method was applied, and the minimum occurrence threshold was set to 5. Forty-nine keywords met this threshold. Before visualization, spelling and hyphenation variants and semantically equivalent terms were consolidated, whereas overly generic, duplicated, or topic-irrelevant terms were excluded. The network and density maps in Figure 1a,b were generated directly using VOSviewer. Generative artificial intelligence (OpenAI ChatGPT, GPT-5 series; OpenAI, San Francisco, CA, USA) was used solely to assist with English-language refinement and the drafting and redrawing of author-developed schematic figures, including layout, typography, and vector-graphic organization.

The resulting maps show that “Ti–6Al–4V alloy” and “ultrasonic surface rolling process” constitute the central material–process core, while “microstructure evolution,” “mechanical properties,” “fatigue,” “residual stress,” “nanocrystallization,” “surface integrity,” and “gradient nanostructure” form the principal mechanism–property cluster. The close association of fatigue-related terms with residual stress, gradient nanostructure, and surface integrity reflects the dominant research focus on fatigue enhancement through coordinated control of the microstructure and stress state. More specialized terms, including “fretting fatigue,” “fretting wear,” “corrosion resistance,” “plasma electrolytic oxidation,” “high-cycle fatigue,” and “crack growth,” indicate the extension of USRP research toward contact damage, environmental degradation, coating–substrate combinations, and failure mechanism analysis. Overall, the maps demonstrate that this field has evolved from establishing the surface-strengthening effects of USRP toward correlating microstructural evolution and residual stress with application-specific service performance.

The annual literature distribution and thematic evolution indicate that research on USRP of titanium alloys has progressed through four stages: methodological foundation, mechanistic expansion, hybrid process development, and data-driven optimization. Before 2016, only a few studies were available, mainly concerning ultrasonic rolling contact, plastic deformation, and numerical prediction of residual stress, which established the methodological basis for subsequent titanium alloy research [25]. From 2016 to 2019, attention shifted to the effects of static load, ultrasonic frequency, and processing passes on surface morphology, hardness, gradient microstructure, residual compressive stress, and fatigue performance, and preliminary mechanisms were proposed for α/β refinement and local phase transformation [3,11,12,13,14,26]. From 2020 to 2024, the material scope expanded to additively manufactured materials, welded joints, coating substrates, and near-net-shaped blades. Electropulsing, temperature assistance, deep cryogenic treatment, and PEO combinations were progressively developed, and the objective moved from isolated surface hardening toward coordinated control of microstructure, stress, morphology, and service performance [20,21,22,23,24,27,28,29,30].

Since 2025, research has increasingly focused on high-strain-rate mechanisms, trajectory coverage, thermo-mechanical coupling, and data-driven optimization. Ultrasonic amplitude affects not only contact load but also low-angle grain boundaries, hardness gradients, residual stress, and fatigue life by changing impact kinetic energy and the surface layer strain rate [19]. Studies of eccentric rolling devices and trajectory interference have introduced processing efficiency, trajectory coverage, and strengthening uniformity as new process evaluation metrics [31,32,33]. Laser-assisted USRP has further revealed competition between thermal softening and mechanical strengthening, showing that excessive heat input may cause surface oxidation, microstructural recovery, and attenuation of strengthening [34]. Meanwhile, neural network-based models and multi-objective surrogate models have been applied to fatigue life prediction for Ti–6Al–4V and USRP parameter optimization for TC4 alloy [35,36]. Notably, a recent study on TC21 titanium alloy found that USRP improved surface integrity and tensile properties but reduced rotating–bending high-cycle fatigue strength by 7.7% [37]. This counterexample demonstrates that USRP strengthening does not necessarily increase monotonically with deformation severity, hardness, or residual compressive stress; it also depends on the mechanical compatibility between the gradient layer and substrate, surface and subsurface defects, and the specific cyclic-loading mode.

## 3. Fundamentals and Process Control of USRP

### 3.1. Working Principle, Equipment Configurations, and Terminological Boundaries

**Figure 2:**
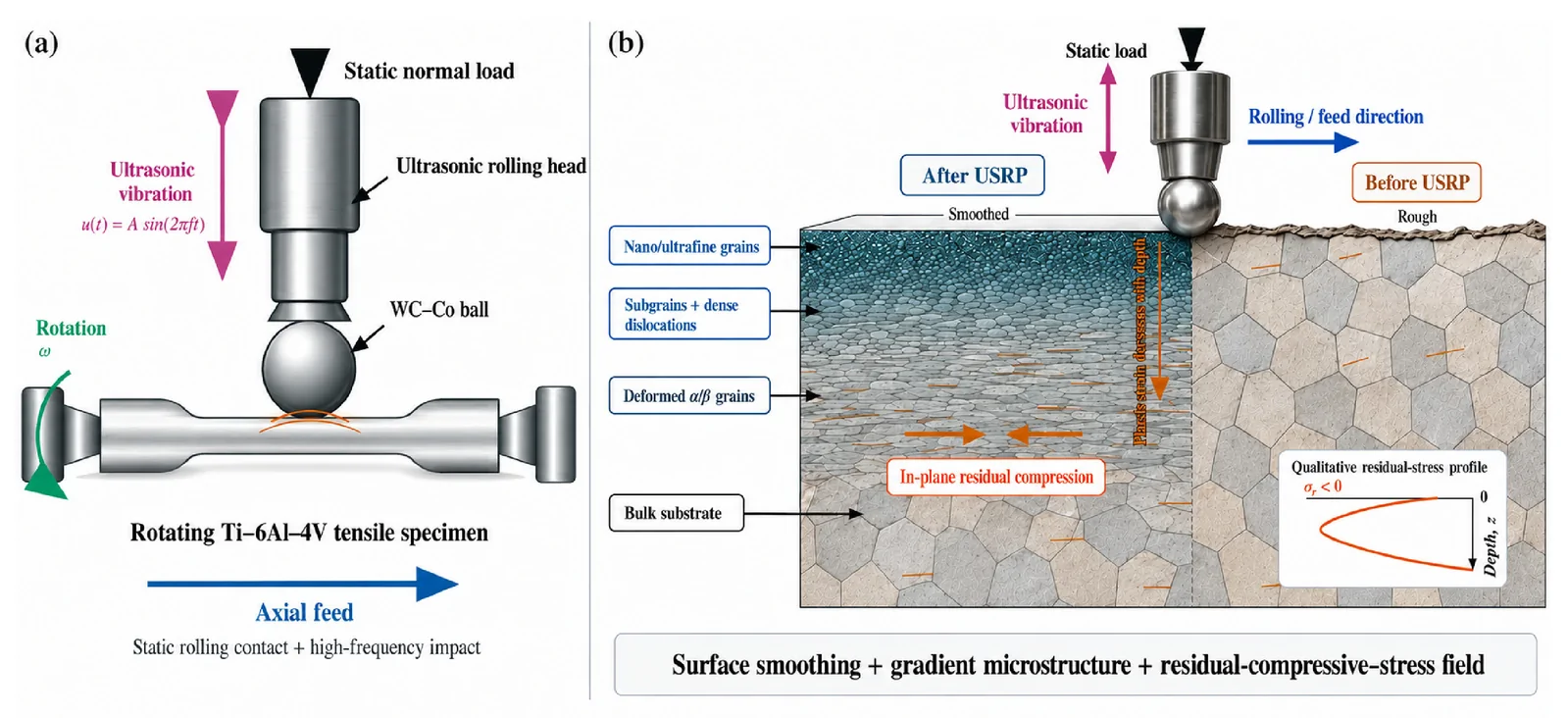
Schematics of USRP applied to (**a**) a rotating cylindrical specimen and (**b**) a plate specimen, illustrating the principal loading components and the resulting one-sided gradient microstructure. Developed based on Refs. [3,4].

USRP applies a static load to the workpiece surface through a rolling head, superimposes high-frequency ultrasonic vibration, and produces a continuous processing trajectory through workpiece rotation or rolling head feed, as shown in Figure 2. Periodic indentation, unloading, and re-indentation by the rolling head promote plastic flow in the surface layer, flatten asperities, and fill valleys, thereby reducing surface roughness. Repeated compressive plastic deformation causes work hardening, microstructural refinement, and residual compressive stress [3,4]. USRP is, therefore, neither a purely ultrasonic impact process nor static rolling but a surface plastic-processing method involving the combined action of static preload, high-frequency vibration, and trajectory motion.

According to workpiece geometry and loading mode, USRP equipment can be classified into lathe-type systems for shafts, mobile systems for planar surfaces, and eccentric rolling systems. An eccentric configuration converts transducer vibration into periodic radial loading, thereby increasing processing efficiency while altering the distributions of contact force and residual stress [31,32]. The terms USRP, URSP, and SUR generally denote closely related techniques, whereas UNSM emphasizes high-frequency point impacts by a spherical indenter along a discrete trajectory. Because these processes differ in indenter motion, contact mode, and the number of impacts per unit area, comparisons among studies should consider equipment configuration, loading form, and processing trajectory rather than classifying techniques solely by their abbreviations.

### 3.2. Contact Dynamics and High-Strain-Rate Deformation Characteristics

The initial static indentation state during USRP can be described by Hertzian contact theory, but ultrasonic vibration produces pronounced time-dependent and nonlinear behavior at the rolling head/workpiece interface. Static load mainly determines the mean indentation depth and plastic zone size, whereas ultrasonic amplitude and frequency govern dynamic contact force, instantaneous impact energy, and loading rate. When the amplitude exceeds a specific dynamic boundary, the rolling head may transition from relatively stable contact to periodic separation, impact, and rebound, causing contact force fluctuations and nonuniform surface deformation [9,31,38,39].

Numerical simulations showed that, at static loads of 900 and 1800 N, the deformation strain rates at the center of the Ti–6Al–4V rolling contact zone can reach approximately 2157 and 4215 s^−1^, respectively, demonstrating that the coupling of static load and dynamic ultrasonic impact produces high-strain-rate plastic deformation in the surface layer [40]. Further studies showed that increasing amplitude raises impact kinetic energy and surface strain rate and modifies the hardness, residual stress, and fatigue responses, although the effect is limited by contact stability and material deformability [19]. The severity of USRP strengthening should, therefore, be described by static indentation, dynamic impact, and repeated action per unit area.

### 3.3. Key Process Parameters and Their Coupling

USRP parameters can be grouped into three categories. The first comprises loading and tool parameters, including static load, amplitude, frequency, and rolling head size and shape. The second comprises motion parameters, including spindle speed, feed, trajectory spacing, coverage, and processing passes. The third comprises interface and material conditions, including lubrication, friction, treatment temperature, initial roughness, and initial microstructure. These parameters are not independent; by altering contact pressure, impact density, strain rate, and accumulated plastic strain, they jointly determine the final surface integrity.

Moderate increases in static load or amplitude generally enlarge the plastically deformed zone and residual compressive stress layer, but excessive amplitude may cause unstable rebound of the rolling head, whereas excessive load may produce material pile-up and surface damage [9,26,31,38,39]. Spindle speed and feed jointly determine the number of impacts per unit length and the degree of trajectory overlap, and their effects are usually non-monotonic. An appropriate spindle speed improves surface morphology and increases hardness and residual compressive stress, but the outcome is controlled jointly by trajectory overlap and the number of impacts per unit area [17,33,41]. Additional processing passes accumulate plastic deformation, but excessive repetition may increase roughness, relax residual stress, or saturate strengthening [18]. Consequently, nominal “optimal parameters” should not be transferred directly between different machines. Instead, process windows should be constructed using energy input per unit area, strain rate, coverage, and repeated passes, and reports should include static load, amplitude, frequency, rolling head dimensions, rotational speed, feed, trajectory spacing, passes, lubrication, and temperature rise.

## 4. Evolution and Control Mechanisms of Surface Integrity

USRP does not regulate the surface integrity of Ti–6Al–4V through a single parameter; rather, surface morphology, gradient microstructure, hardness, and residual stress evolve synergistically. Because plastic strain and strain rate decay gradually from the surface toward the substrate, all these characteristics exhibit pronounced depth gradients, although their peak positions and effective ranges do not necessarily coincide.

### 4.1. Surface Morphology and Defect Control

During USRP, static indentation and high-frequency impact by the rolling head drive lateral plastic flow of surface material, flattening machining asperities and filling adjacent microvalleys, thereby reducing turning marks, grooves, and local pits. For turned TC4 alloy, an appropriate USRP treatment reduced Ra from 1.892 μm to 0.05–0.20 μm, with a minimum of 0.052 μm [7]. Trajectory interference experiments further showed that increasing coverage improves the uniformity of material flow and reduces Ra to 0.022 μm by filling microvalleys and suppressing adhesive wear [33].

**Figure 3:**
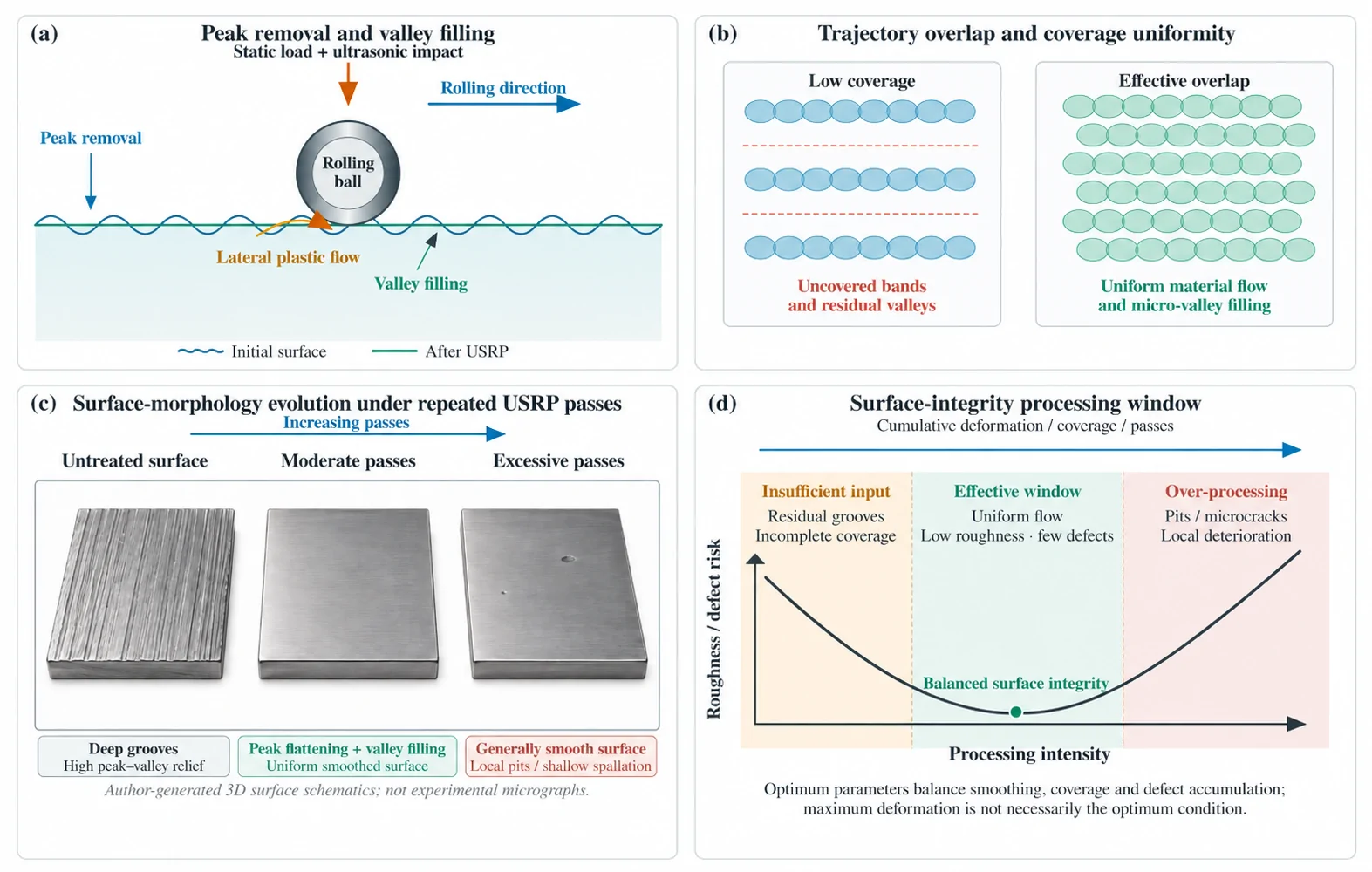
Regulation of surface morphology and defect evolution in Ti–6Al–4V alloy by USRP: (**a**) asperity flattening and valley-filling mechanisms under the rolling head; (**b**) effect of trajectory coverage on the uniformity of surface material flow; (**c**) schematic illustration of the evolution of surface morphology and roughness with increasing processing passes, drawn based on the underlying mechanisms; and (**d**) surface integrity process window jointly governed by accumulated deformation, coverage, and processing passes. Author-developed schematic based on Refs. [18,33].

The decrease in roughness does not continue monotonically with the number of processing passes. Multipass experiments showed that additional passes progressively eliminate original machining marks; however, when the number of passes reaches 15–20, pits and microcracks reappear, and the roughness increases [18]. These pass- and coverage-dependent mechanisms are summarized in Figure 3, including peak removal and valley filling, trajectory overlap-controlled surface uniformity, the evolution of surface morphology with increasing passes, and the transition from insufficient processing to an effective window and eventual over-processing. The central requirement in surface morphology control is, therefore, to achieve sufficient and uniform plastic flow while avoiding surface plastic exhaustion, material spalling, and defect accumulation caused by overprocessing. Initial morphology, indentation depth, trajectory coverage, processing passes, and lubrication state should be evaluated as interrelated parameters rather than judging treatment effectiveness solely from minimum roughness.

### 4.2. Gradient Microstructure and Phase-Transformation Behavior

Because contact stress, plastic strain, and strain rate decrease with depth, USRP generally produces a gradient structure consisting of a surface nanocrystalline or nanolamellar zone, a subsurface ultrafine-grained zone, a deformed coarse-grained zone, and the substrate [6,11,12]. For the α phase, a gradient microstructure with a total thickness exceeding 400 μm has been observed, including a near-surface equiaxed nanograined layer, nanolamellae, ultrafine lamellae, refined grains, and a low-strain coarse-grained region. Refinement is mainly driven by dislocation slip, entanglement, and rearrangement. Coarse hcp-α grains are first elongated and then progressively transformed into near-equiaxed nanograins through longitudinal subdivision and transverse fragmentation, accompanied by dynamic recovery [11].

The refinement path of the β phase differs from that of the α phase. Dislocations first nucleate near α/β interfaces and propagate into the β phase, subsequently forming dislocation arrays, dislocation bands, and low-angle grain boundaries. With strain accumulation, low-angle boundaries gradually transform into high-angle boundaries, and the original β grains are converted into equiaxed nanograins through longitudinal subdivision and transverse fragmentation. The reported β grain size decreases progressively from approximately 0.76 μm in the interior to approximately 36.5 nm at the surface, without observable deformation twinning in the β phase [12].

**Figure 4:**
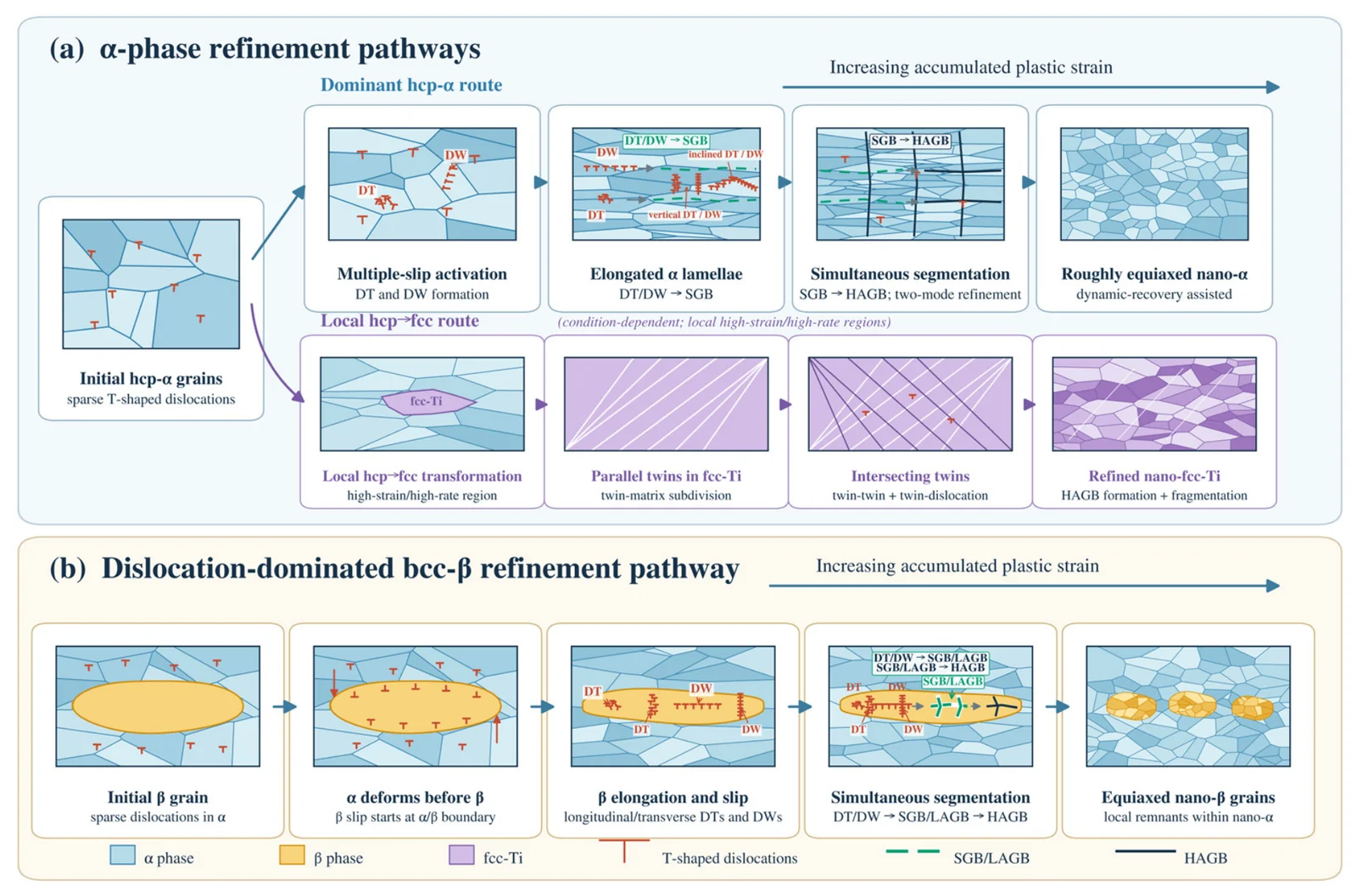
Schematic of phase-dependent grain refinement in USRP-treated Ti–6Al–4V alloy: (**a**) dominant dislocation-mediated refinement of hcp-α and a local hcp → fcc transformation–refinement route; and (**b**) dislocation-dominated refinement of bcc-β through DT/DW accumulation, SGB/LAGB formation, boundary transformation, and grain fragmentation. DT, dislocation tangle; DW, dislocation wall; SGB, subgrain boundary; LAGB, low-angle grain boundary; HAGB, high-angle grain boundary. Developed by the authors based on Refs. [11,12].

Several studies have observed an hcp-Ti → fcc-Ti transformation in the USRP-treated layer and identified twins and twin–dislocation interactions within fcc-Ti [11,13]. In the transformed fcc-Ti regions, parallel twins initially subdivide the grains into twin–matrix lamellae. With increasing deformation, twin–twin intersections and twin–dislocation interactions promote high-angle-boundary formation, longitudinal splitting, transverse breakdown, and eventual nanograin formation [11]. However, fcc-Ti formation depends on the initial microstructure, local strain, strain rate, and phase-boundary state and should not be regarded as a universal mechanism in all USRP-treated Ti–6Al–4V alloys. These phase-dependent refinement pathways are summarized schematically in Figure 4. In additively manufactured materials, the original lamellar microstructure, columnar prior β grains, texture, and pores additionally modify strain partitioning and refinement paths. USRP can transform coarse lamellae into ultrafine lamellae, equiaxed ultrafine grains, and nanograins and can densify the near-surface microstructure [20,27,42].

### 4.3. Work Hardening and Hardness Gradient

Hardening of the USRP-treated layer arises mainly from dislocation strengthening, grain refinement, subgrain boundary formation, and obstruction of dislocation motion by α/β phase boundaries. Because deformation severity normally decreases from the surface toward the interior, hardness generally decreases gradually from the strengthened layer to the substrate [6,7,18]. Increasing static load, amplitude, or processing passes can increase hardness and deepen the hardened layer within a certain range, whereas dynamic recovery, local heating, and surface damage caused by overprocessing may weaken the hardening effect.

**Figure 5:**
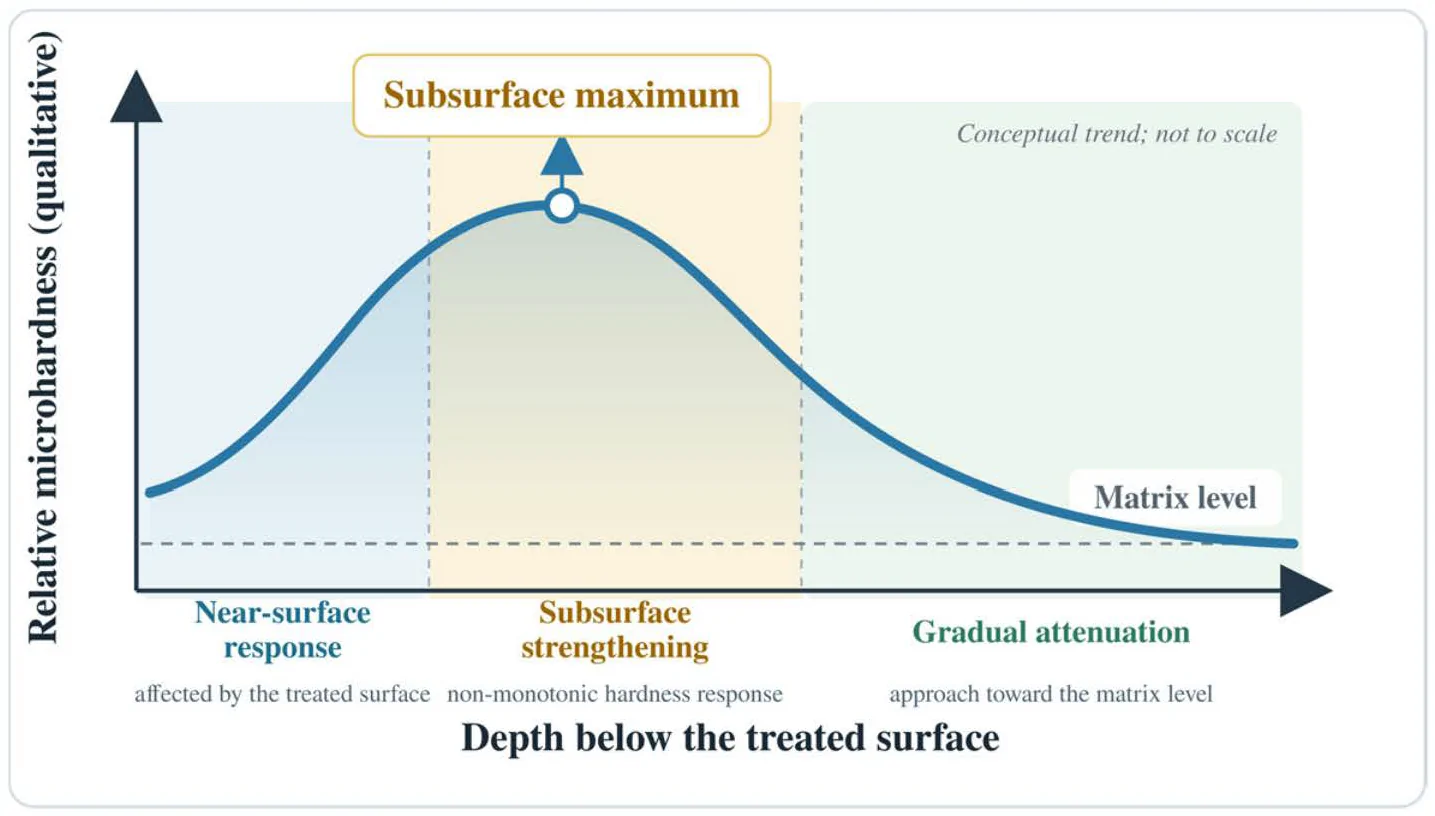
Conceptual schematic of the non-monotonic microhardness depth response after USRP, characterized by a subsurface maximum followed by gradual attenuation toward the matrix level. The qualitative trend was summarized from Ref. [28].

Importantly, the maximum hardness is not necessarily located at the outermost surface. As shown in Figure 5, the hardness of USRP-treated Ti–6Al–4V exhibits a non-monotonic depth profile, reaching a subsurface maximum at approximately 50 μm before gradually decreasing toward the substrate level [19,28]. Further quantitative work showed that, in some USRP specimens, the maximum plastic deformation and highest dislocation accumulation occur below the surface. Dislocation pile-up contributes more to hardening than grain refinement alone, thereby producing a “subsurface hardness peak” [28]. Hardness gradients should, therefore, be interpreted in conjunction with grain size, dislocation density, local misorientation, and the plastic strain field; the finest surface grains do not necessarily correspond to the highest hardness. Indentation load, measurement spacing, and criteria for defining hardened layer depth should also be standardized to improve comparability among studies.

In addition to producing a depthwise hardness gradient, partial surface strengthening can introduce in-plane mechanical heterogeneity. Wang et al. used partial ultrasonic surface rolling (PUSRP) to construct a radial–axial bidirectional heterogeneous structure in TC4 alloy. Coordinated deformation between strengthened and unstrengthened regions improved the overall strength–ductility balance [43]. Thus, the control variables of USRP include not only load and processing passes but also the area fraction and spatial arrangement of the strengthened regions.

### 4.4. Formation, Distribution, and Stability of Residual Compressive Stress

**Figure 6:**
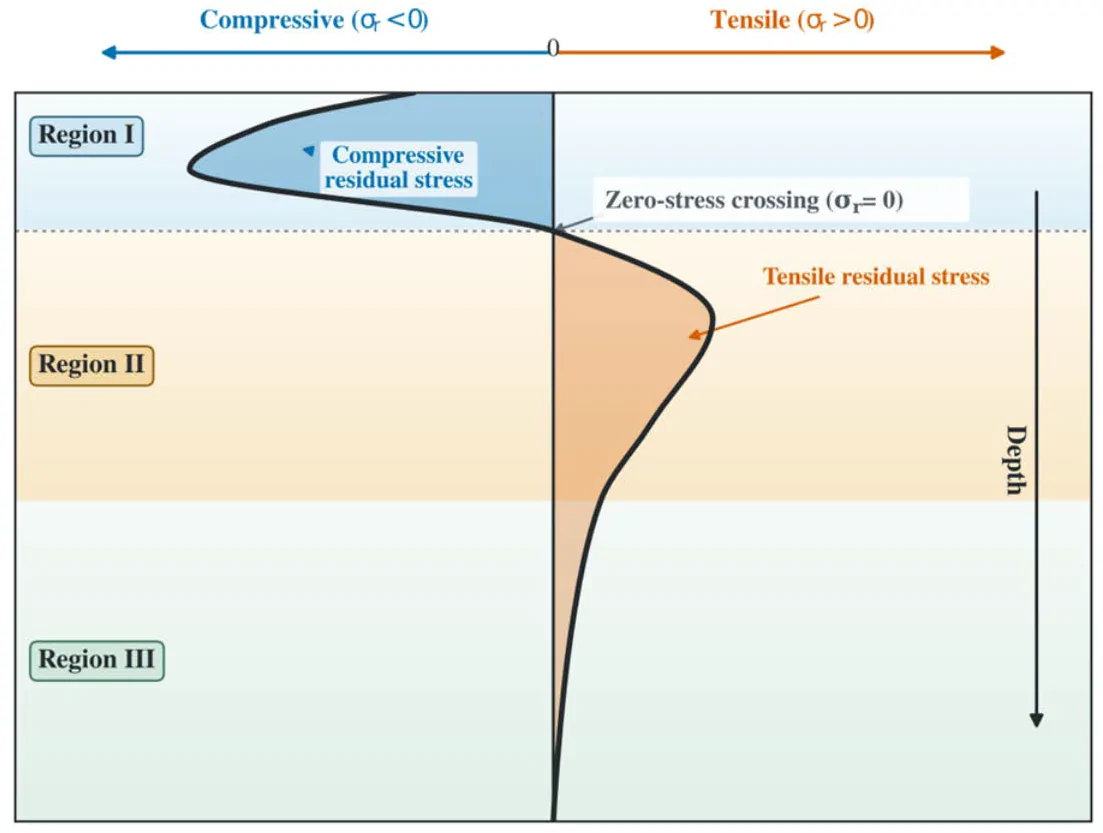
Schematic depth-dependent residual stress distribution in USRP-treated Ti–6Al–4V alloy. The blue and orange areas represent compressive and tensile residual stresses, respectively. Region I is dominated by compressive residual stress, Region II contains balancing tensile residual stress, and Region III represents stress attenuation toward zero. The boundary between Regions I and II marks the zero stress crossing (σ_r_ = 0). Conceptual schematic developed by the authors based on the residual stress partitioning mechanism reported in Ref. [39].

Nonuniform elastoplastic deformation induced by USRP is the primary origin of residual compressive stress. Under normal impact and tangential rolling, the surface material undergoes plastic extension while the less-deformed interior constrains it. After unloading, incompatible elastic recovery between the surface layer and substrate leaves residual compressive stress in the treated region. As schematically illustrated in Figure 6, the residual compressive stress generally reaches its maximum at or beneath the treated surface, subsequently decreases with depth, and may transition into a balancing tensile-stress region before gradually attenuating toward zero [39,44].

The residual stress field is also governed by static load, amplitude, passes, coverage, rolling head geometry, and component curvature. Multipass experiments showed that, after five passes, residual compressive stress of approximately −390 MPa was retained at a depth of 500 μm. When the number of passes increased to 20, the residual compressive stress at 200 μm was only approximately −205 MPa, indicating that overprocessing may cause local stress relaxation [18]. Low-plasticity ultrasonic rolling of a near-net-shaped blade produced a residual compressive stress layer approximately 70 μm deep [24], further demonstrating that stress depths measured on plates or cylindrical bars cannot be extrapolated directly to thin-walled complex components.

Residual stress assessment should, therefore, report not only the maximum surface value but also the stress direction, peak position, effective depth, and treatment uniformity. More importantly, relaxation under cyclic loading, thermal exposure, and corrosive environments should be examined. Residual compressive stress is not an invariant material property; its practical contribution depends on the spatial matching between the stress field and service loading and on its long-term stability.

## 5. Service Performance and Underlying Mechanisms

The influence of USRP on service performance is not a simple consequence of increased surface hardness but depends on the synergy among surface morphology, gradient microstructure, work hardening, and residual compressive stress. Different service conditions also have different sensitivities to surface integrity parameters. Conventional fatigue is controlled mainly by surface defects and effective tensile stress; fretting fatigue additionally involves stress concentration at contact edges and wear damage; and rolling contact fatigue is closely related to cyclic subsurface shear stress. Wear and corrosion performance are additionally affected by surface films and environmental media.

### 5.1. Fatigue Performance and Crack Initiation and Propagation

For conventional fatigue, USRP first reduces geometric notches such as machining grooves and pits through rolling-induced smoothing, thereby decreasing the probability of surface crack initiation. Surface grain refinement and work hardening increase resistance to cyclic plastic deformation, whereas residual compressive stress reduces the effective tensile stress and crack tip driving force generated by external loading, thus delaying short-crack initiation and early propagation [3,14]. In Ti–6Al–4V, USRP increased the fatigue limit corresponding to 10^7^ cycles from 500 to 610 MPa; at a maximum stress of 700 MPa, the fatigue life of the treated specimen was approximately 23.5 times that of the substrate [14]. Factor separation experiments further indicated that residual compressive stress is the principal contributor to fatigue life improvement, whereas gradient microstructure and work hardening act synergistically mainly by increasing resistance to crack initiation [3,14].

USRP fatigue strengthening has an appropriate deformation window. Increasing the number of passes generally deepens the plastically deformed and hardened layers, but overprocessing may cause surface spalling, microcracking, and local residual stress relaxation, such that fatigue performance no longer increases monotonically with deformation severity [16,18]. Under a static load of 550 N, five-pass treatment produced a fatigue life of 3.29 × 10^5^ cycles at 600 MPa, approximately eight times that of the untreated specimen, and shifted the crack source from the surface to a subsurface position approximately 180 μm deep. When the number of passes was increased to 20, fatigue life decreased even though surface hardness and deformation layer depth continued to increase [18]. Assessment of fatigue resistance after USRP must, therefore, consider surface defects, the effective depth of the residual compressive stress layer, and deformation compatibility between the gradient layer and substrate, rather than relying only on surface hardness or maximum residual compressive stress.

**Figure 7:**
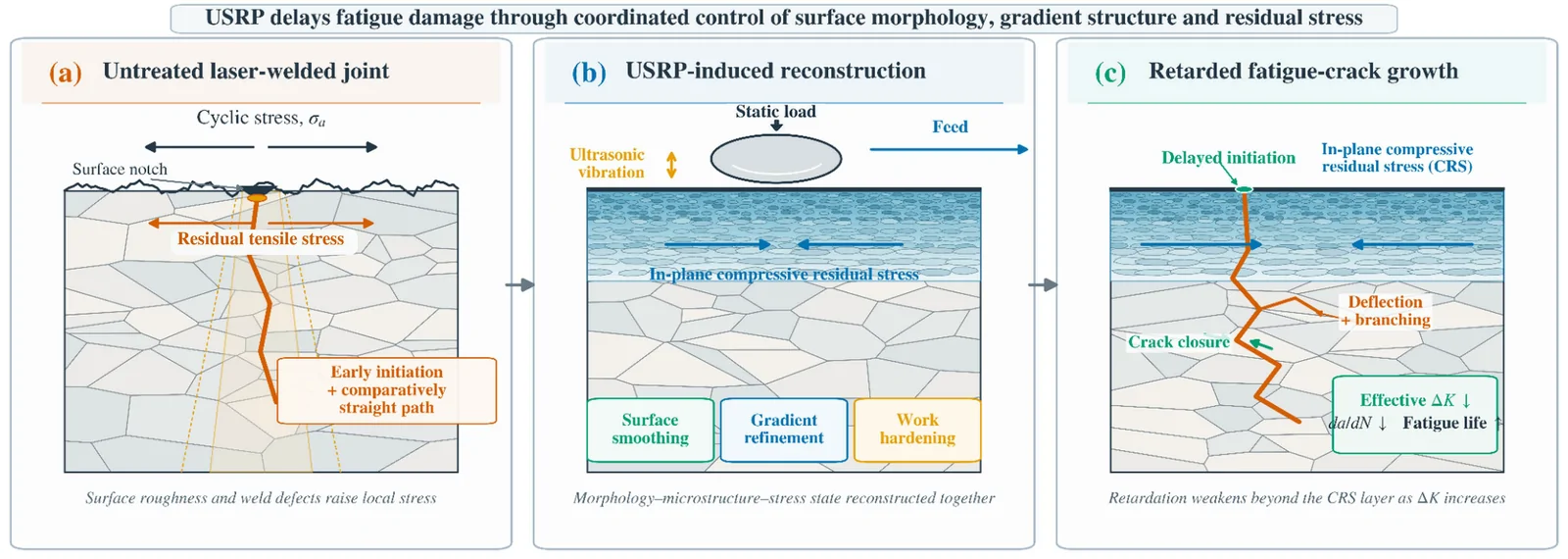
Schematic of fatigue-strengthening mechanisms in USRP-treated Ti–6Al–4V laser-welded joints: (**a**) surface irregularities, weld defects, and tensile residual stress promote early crack initiation in the untreated joint; (**b**) USRP reconstructs surface integrity through surface smoothing, gradient refinement, work hardening, and in-plane compressive residual stress; and (**c**) crack closure, deflection, and branching reduce the effective ΔK and retard crack growth. This effect weakens beyond the compressive stress-affected layer. Author-developed schematic based on Ref. [21].

USRP can also improve the fatigue performance of welded joints and coated components. USRP can also improve the fatigue resistance of Ti–6Al–4V laser-welded joints. In untreated joints, surface irregularities, weld defects, and local residual tensile stress increase the effective crack tip driving force, thereby promoting early surface crack initiation and comparatively straight propagation. USRP smooths the weld surface, produces a gradient-refined and work-hardened layer, and converts the near-surface residual stress state from tensile to compressive. The resulting in-plane compressive residual stress promotes crack closure and reduces the effective stress intensity factor range, whereas the refined and heterogeneous subsurface microstructure facilitates crack deflection, branching, and a more tortuous propagation path. These mechanisms act synergistically to retard crack growth, particularly during crack initiation and stable propagation, as summarized in Figure 7 [21]. As the crack extends beyond the compressive residual stress-affected layer and the crack-driving force increases, the retardation effect gradually weakens. In PEO-coated systems, USRP pretreatment improves the substrate surface condition and retains a deep residual compressive stress layer. With a PEO coating approximately 20 μm thick, the fatigue life of the hybrid-treated specimen at 600 MPa was approximately 14 times that of the PEO-only specimen [22].

USRP does not necessarily improve fatigue performance in every titanium alloy or under every loading mode. A recent study on TC21 alloy showed that USRP improved roughness, residual compressive stress, and tensile properties but reduced rotating–bending high-cycle fatigue strength by 7.7%. This reduction was attributed to interfacial stress concentration and local deformation mismatch caused by a steep mechanical property gradient between the strengthened layer and substrate [37]. This counterexample demonstrates that fatigue-resistant surface design should seek a continuous and stable gradient structure compatible with the substrate rather than maximizing the degree of surface nanocrystallization.

### 5.2. Fretting Fatigue and Rolling Contact Fatigue

Fretting fatigue results from the combined action of cyclic loading, contact pressure, and small-amplitude relative slip, and cracks usually initiate at wear pits or local adhesion zones near contact edges. Residual compressive stress introduced by USRP reduces the maximum principal tensile stress in the contact region and the normal crack-opening driving force and modifies the local multi-axial stress state, thereby delaying the initiation and propagation of fretting cracks. Its effect on shear-dominated damage should be evaluated using a critical plane stress or equivalent damage parameter. Surface hardening and grain refinement additionally reduce adhesion, ploughing, and local plastic accumulation. USRP produced a residual compressive stress field approximately 530 μm deep with a maximum of approximately −930 MPa in Ti–6Al–4V, increasing the fretting fatigue limit by 72.7% [45]. In another study, the fretting fatigue strength increased by 113.6%, the crack path changed from relatively straight to tortuous, and residual compressive stress contributed more than the gradient nanostructure and work hardening [46].

The influence of residual compressive stress is generally more pronounced during fretting crack propagation than during initial wear pit formation. Finite element simulations and interrupted tests showed that the maximum equivalent damage stress, dissipated energy, and relative slip in the contact region can identify the crack initiation site, whereas deep residual compressive stress mainly reduces the crack growth driving force [15]. Excessive surface deformation may also cause microstructural coarsening, local crystallization, or rapid residual stress relaxation during fretting cycles; fretting fatigue performance is, therefore, likewise deformation severity dependent [47]. Laser-assisted USRP increased the fretting fatigue life of TC11 alloy at a maximum cyclic stress of 750 MPa to 6.4 times that of the substrate by improving surface flatness, residual stress stability, and frictional conditions. This demonstrates the potential of thermally assisted processing, although thermal softening and oxidation must still be controlled [48].

**Figure 8:**
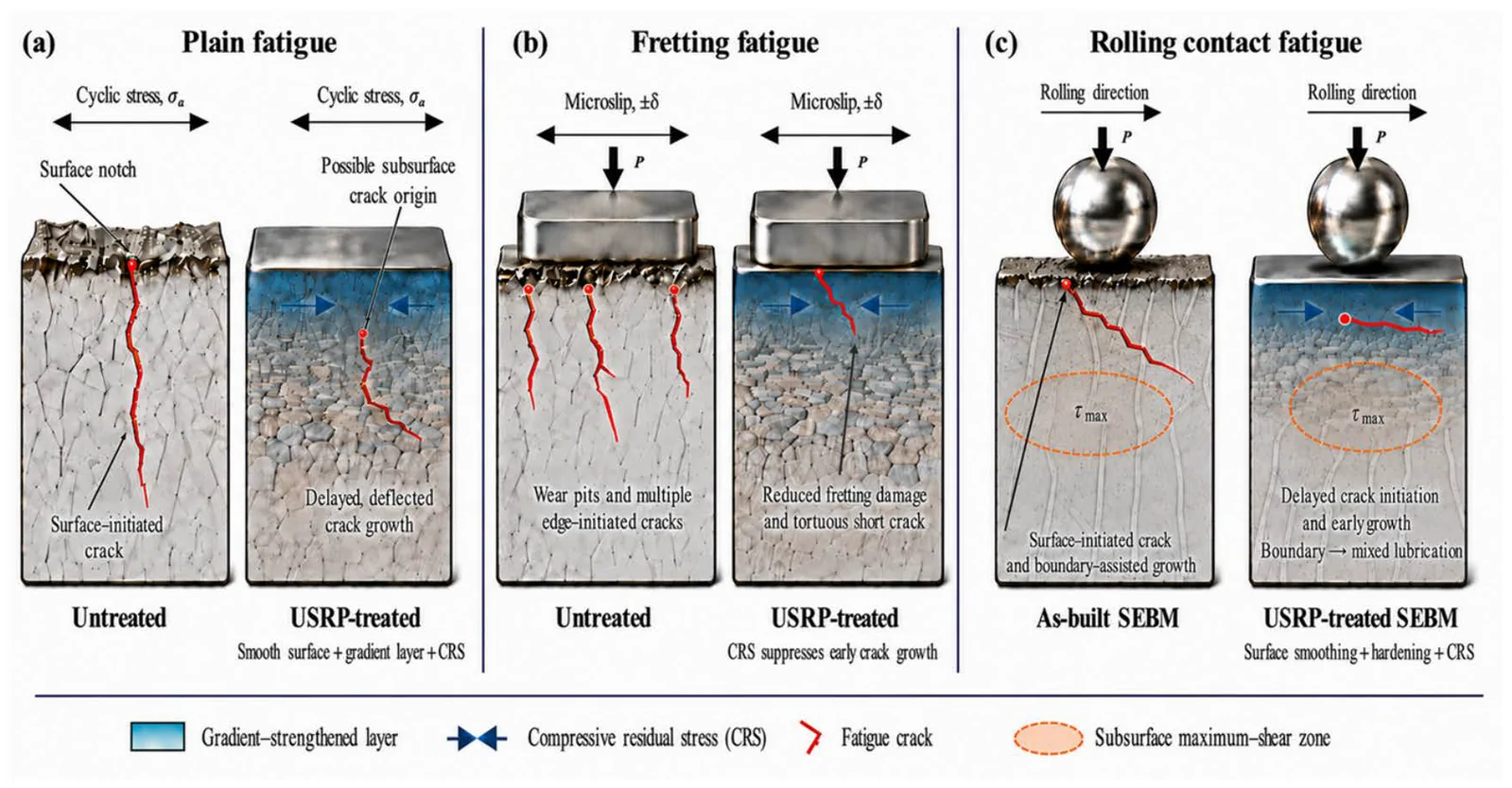
Schematic of fatigue damage mechanisms in titanium alloys under three cyclic-loading/contact conditions before and after USRP: (**a**) conventional fatigue; (**b**) fretting fatigue; and (**c**) rolling contact fatigue. Developed based on Refs. [18,29,30,46].

In rolling contact fatigue, crack initiation and propagation are jointly governed by surface defects, lubrication, and cyclic subsurface shear stress. For selective electron-beam-melted Ti–6Al–4V, build orientation resulted in 54% higher rolling contact fatigue performance in the xoz direction than in the xoy direction, and USRP further increased the mean rolling contact fatigue life by approximately 30% [29]. Strengthening was attributed to surface densification, work hardening, nanocrystallization, residual compressive stress, and a transition from boundary to mixed lubrication. When induction heating-assisted USRP was performed at 650 °C, the surface hardness of additively manufactured Ti–6Al–4V increased by 33% relative to the untreated condition, and the mean rolling contact fatigue life increased by 196% [30]. However, the oxide mixture layer generated during high-temperature treatment and internal pores originating from additive manufacturing cannot be completely eliminated, and surface strengthening cannot replace control of internal metallurgical defects. The mode-dependent damage processes and corresponding USRP strengthening mechanisms under conventional fatigue, fretting fatigue, and rolling contact fatigue are compared schematically in Figure 8.

### 5.3. Wear, Erosion, and Corrosion Performance

**Figure 9:**
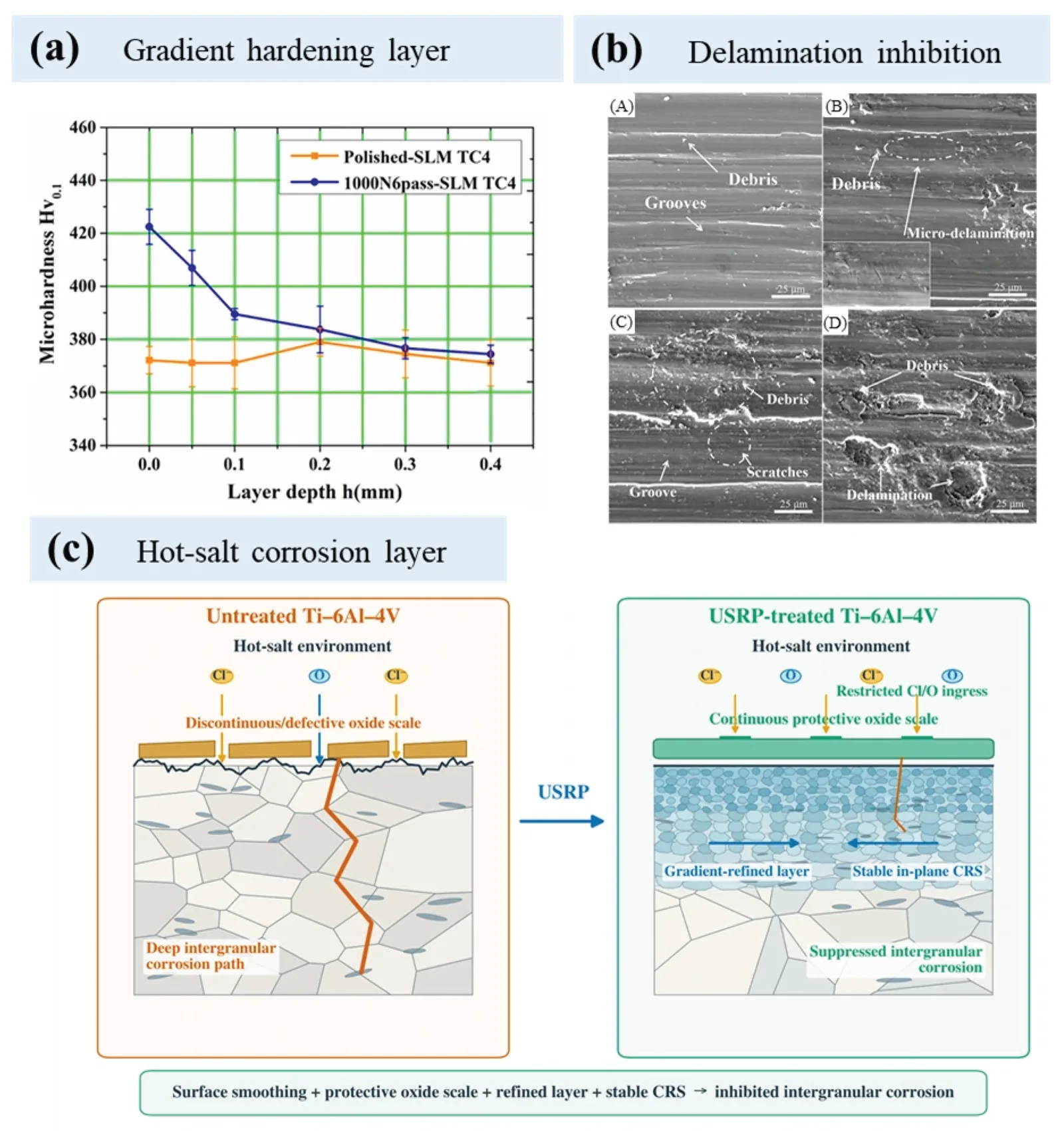
Experimental evidence and mechanisms underlying the improved wear and hot salt corrosion resistance of Ti–6Al–4V after USRP: (**a**) depth-dependent microhardness profiles of polished and USRP-treated SLM Ti–6Al–4V; (**b**) representative worn-surface morphologies showing that USRP delays micro-delamination and delamination damage, (**A**) 3; (**B**) 6; (**C**) 9 and (**D**) 15 min; and (**c**) schematic illustrating the synergistic restriction of Cl/O ingress and intergranular corrosion by surface smoothing, gradient refinement, a protective oxide scale, and stable in-plane compressive residual stress. Panels (**a**,**b**) were adapted from Wang et al. [42], © 2017 the authors, licensed under CC BY 4.0 (https://creativecommons.org/licenses/by/4.0/); the original panels were cropped, relabeled, and rearranged for clarity. Panel (**c**) was developed by the authors based on Ref. [5].

The improvement in wear resistance produced by USRP arises mainly from surface smoothing, work hardening, gradient microstructure, and residual compressive stress. Higher surface hardness reduces counterbody penetration and suppresses ploughing, while microstructural refinement and residual compressive stress retard the coalescence of subsurface cracks and the formation of delamination. In selective laser-melted Ti–6Al–4V, USRP reduced the dry-sliding coefficient of friction from 0.74 to 0.64 and the wear volume from 0.205 to 0.195 mm^3^. Rather than completely changing the final wear mode, it mainly delayed the onset of adhesive wear and fatigue delamination [42]. The corresponding depth-dependent microhardness profiles and representative worn-surface morphologies are shown in Figure 9a,b. For HIP Ti–6Al–4V, ultrasonic frequency and static load jointly affected the hardened layer and fretting wear behavior, with 30 kHz and 900 N producing a favorable overall surface condition [26].

Results for related titanium alloys under extreme environments further show that wear resistance depends on the thermal stability and environmental adaptability of the strengthened layer. After USRP of TC11 alloy, surface roughness decreased by 80.4%, while hardness and residual compressive stress increased by 21.7% and 97.1%, respectively. Lower coefficients of friction and wear losses were obtained at room temperature, −60 °C, 400 °C, and under seawater corrosion–wear conditions, and the dominant wear mode changed from adhesive wear to relatively mild abrasive wear [49]. Under solid-particle erosion, increasing the static load enhanced erosion resistance in Ti–6Al–4V, with a more pronounced improvement at low impact angles. However, erosion rate, failure of the strengthened layer, and residual stress evolution after prolonged erosion have not yet been systematically quantified [50].

The effect of USRP on electrochemical corrosion performance depends strongly on the medium and processing parameters. Moderate grain refinement can increase passive film nucleation sites, and a smoother surface facilitates the formation of a continuous and dense protective film. Conversely, excessive defect density, surface cracking, or locally nonuniform plastic deformation may increase surface chemical activity. For Ti–6Al–4V treated with different numbers of USRP passes, the charge transfer resistance in 3.5 wt.% NaCl solution differed only slightly overall, whereas USRP-treated specimens exhibited higher charge transfer resistance in 1 mol/L HCl solution, with the five-pass specimen showing the densest passive film and highest corrosion resistance [18]. It is, therefore, inappropriate to state generally that “finer grains always provide higher corrosion resistance.”

In a solid-NaCl hot salt environment at 400 °C, the rough surface, local defects, and complete relaxation of residual compressive stress produced by shot peening prevented any improvement in the hot salt corrosion resistance of Ti–6Al–4V. In contrast, USRP promoted the formation of a protective oxide film and suppressed intergranular corrosion through lower roughness, a more thermally stable deep residual compressive stress field, and refined microstructure [5]. A study on TC11 alloy further showed that USRP can maintain relatively stable residual compressive stress under coupled thermal–mechanical–salt conditions at 500 °C and promote the formation of a dense amorphous–nanocrystalline composite oxide film, thereby retarding the diffusion of Cl and O into the substrate and increasing resistance to hot salt stress corrosion cracking [51]. The critical factors in a corrosive environment are, therefore, not the initial peak residual compressive stress but the integrity of the surface film and the stability of residual compressive stress under the combined effects of temperature, loading, and corrosion.

## 6. Hybrid USRP Control Technologies

Conventional USRP relies mainly on static rolling and high-frequency impacts to induce surface plastic deformation. Further increases in load, amplitude, or processing passes may increase deformation severity but may also cause surface damage, work-hardening saturation, and residual stress relaxation. The central purpose of hybrid USRP is, therefore, not simply to superimpose energy but to use electrical, thermal, or chemical surface modification to change instantaneous deformability, defect evolution, and interfacial load-bearing behavior, thereby balancing promotion of plastic deformation against retention of strengthening.

### 6.1. Electropulsing-Assisted USRP

Electropulsing-assisted USRP includes EP-USRP, DC-USRP, and EUSRP. Joule heating generated by the current reduces the instantaneous flow stress of Ti–6Al–4V and promotes dislocation motion and surface plastic flow, while electron–dislocation interactions may produce an electroplastic effect [52,53]. Existing experiments, however, generally cannot completely separate thermal effects from nonthermal electroplasticity; it is, therefore, not rigorous to attribute all microstructural evolution to the “electron wind force.”

For selective laser-melted Ti–6Al–4V, the wear rate of the DC-USRP specimen under lubricated conditions was 1 × 10^−6^ mm^3^·N^−1^·m^−1^, lower than the 5 × 10^−6^ mm^3^·N^−1^·m^−1^ of the conventional USRP specimen, indicating that current assistance improves plastic flow, surface defects, and the load-bearing capacity of the gradient hardened layer [54]. Recent work has further coupled pulsed current with ultrasonic rolling to control fretting wear in Ti–6Al–4V and showed that current-assisted plastic deformation, the surface-hardening state, and friction interface evolution jointly determine friction reduction and wear resistance [55]. Increasing current does not necessarily improve fatigue performance. Excessive Joule heating promotes dislocation recovery, reduces work hardening, and may weaken both the magnitude and cyclic stability of residual compressive stress.

**Figure 10:**
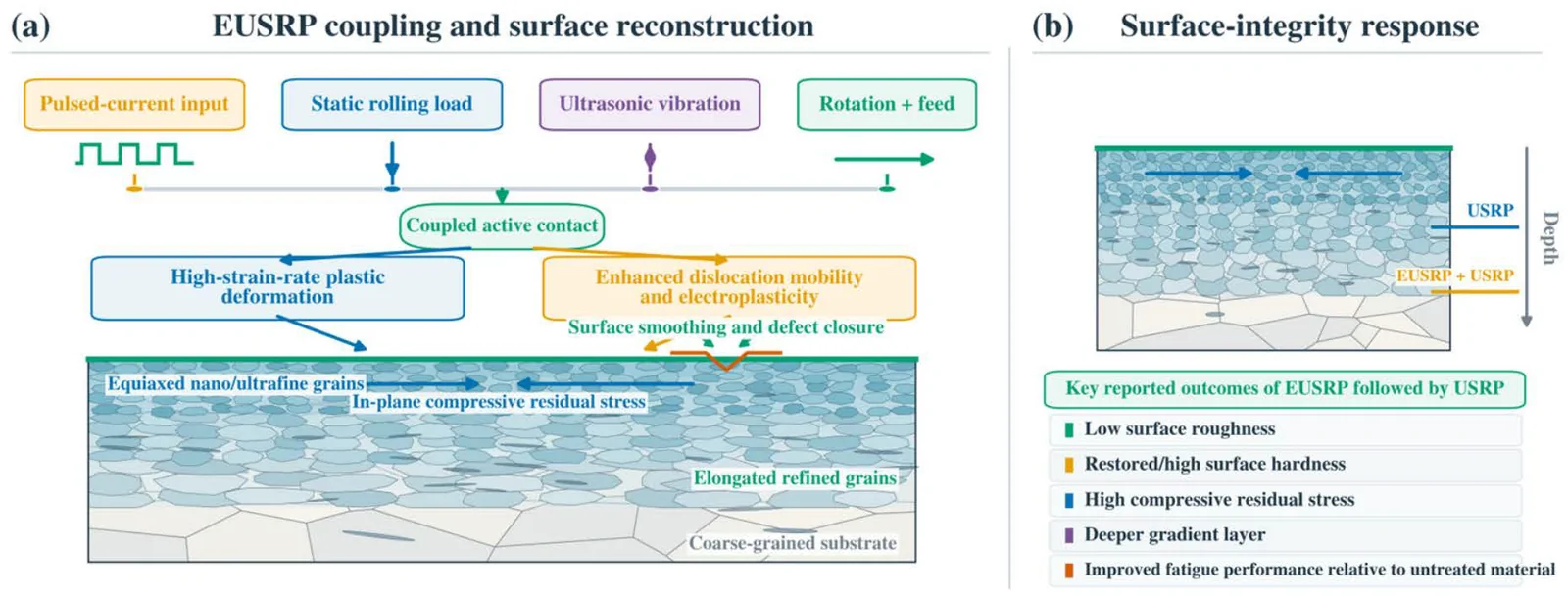
Schematic of EUSRP followed by USRP for surface modification of Ti–6Al–4V alloy: (**a**) coupled pulsed-current input, static rolling load, ultrasonic vibration, rotation and feed, and the associated high-strain-rate deformation and electroplasticity; and (**b**) key reported outcomes, including low surface roughness, restored/high surface hardness, high compressive residual stress, a deeper gradient layer, and improved fatigue performance relative to untreated material. The schematic was developed by the authors based on the mechanistic evidence reported in Ref. [23].

Sequential hybrid processing offers a possible solution to this conflict. Sun et al. first applied 200 A electropulsing-assisted rolling to promote plastic flow and enlarge the deformation zone and then employed conventional USRP to restore surface hardening and compressive residual stress [23]. The coupled processing inputs and the associated high-strain-rate deformation and electroplastic effects are illustrated in Figure 10a. Although the electropulsing-assisted stage produced a gradient deformation layer approximately three times as deep as that produced by conventional USRP, the sustained current and associated skin effect were accompanied by a reduction in surface hardness. The subsequent USRP step reduced surface roughness, restored high surface hardness and high compressive residual stress, and retained a gradient deformation layer approximately 400 μm deep. These key reported outcomes, together with the improvement in fatigue performance relative to the untreated material, are summarized in Figure 10b. At a stress level of 780 MPa, the fatigue life of the sequentially treated specimen was approximately 25 times that of the untreated specimen [23]. Nevertheless, its fatigue limit was not the highest among all treated conditions, indicating that maximizing deformation layer depth or surface hardening alone does not necessarily ensure the optimum cyclic response. The role of the pulsed current skin effect in the surface strengthening and microstructural evolution of Ti–6Al–4V ELI remains insufficiently understood. Control variables for electropulsing-assisted processing should, therefore, include not only peak current but also pulse width, duty cycle, current application sequence, and the severity of subsequent mechanical strengthening. The optimization objective should shift from maximizing deformation layer depth to obtaining a stable combination of gradient microstructure, hardness, and residual stress.

### 6.2. Temperature-Assisted USRP

Temperature has a characteristic dual effect on USRP. Moderate heating reduces deformation resistance, activates additional slip systems, and increases the plastic deformation depth, whereas excessive temperature causes dislocation recovery, grain growth, surface oxidation, and attenuation of residual compressive stress. Temperature-assisted USRP, therefore, has an effective temperature window that depends on the initial microstructure, heat source type, and processing duration.

After ultrasonic rolling of hot isostatically pressed Ti–6Al–4V at 120 °C, surface hardness and residual stress reached 414 HV and −491 MPa, respectively, exceeding the 323 HV and −235 MPa of the untreated specimen. Nevertheless, its wear resistance remained lower than that of the room temperature USRP specimen, indicating that heating promoted plastic flow while weakening cold work hardening [56]. Another study showed that heating-assisted ultrasonic rolling at 140 °C produced a surface hardness of 495 HV, approximately 70 HV higher than that after room temperature rolling, but the residual compressive stress was lower and surface roughness slightly higher than under room temperature treatment [57]. These findings demonstrate that hardness, roughness, and residual stress do not respond synchronously to temperature and that no single metric can define the optimal temperature.

Temperature assistance has a more pronounced effect on additively manufactured surfaces. After induction heating-assisted USRP at 650 °C of selective electron beam-melted Ti–6Al–4V with an initially rough as-built surface, Ra and Rz decreased by approximately 96% and 98%, respectively, surface hardness increased by approximately 33%, and mean rolling contact fatigue life increased by approximately 196% [30]. Under these conditions, thermal softening facilitates plastic filling of the original asperities and valleys and blunts surface defects; this role differs from conventional thermally assisted strengthening of a smooth forged material.

Laser heating is local, rapid, and controllable. A study on TC11 showed that, at 750 MPa, the fretting fatigue life of the laser-assisted USRP specimen was approximately 6.4 times that of the substrate, owing to improved surface flatness, activation of slip systems, and enhanced residual stress stability [48]. Recent research on laser-assisted USRP of Ti–6Al–4V further confirmed a critical processing window for the transition from strengthening to softening: appropriate heat input promotes dislocation–twin cooperative deformation, whereas excessive heat input causes dislocation recovery and brittle-oxide formation [34]. Combining deep cryogenic treatment with USRP changes the initial phase constitution and defect state and thereby affects subsequent plastic deformation and hardening, but systematic comparisons of process sequence and low-temperature microstructural stability remain lacking [58].

Temperature-assisted USRP must, therefore, distinguish at least two issues: regulation of plastic deformability by temperature during processing and the stability of the resulting gradient microstructure and residual compressive stress at service temperature. Reporting only post-treatment room temperature hardness or surface roughness is insufficient to evaluate the engineering effectiveness of thermally assisted processing.

### 6.3. USRP Combined with Coatings and Surface-Modification Technologies

The purpose of combining USRP with coating technologies is to establish a multilayer surface architecture comprising a functional coating, a gradient-strengthened substrate, and a deep residual compressive stress field. The coating provides wear resistance, corrosion resistance, and environmental barrier functions, whereas the USRP-treated substrate supports the coating and retards the propagation of fatigue cracks into the interior.

**Figure 11:**
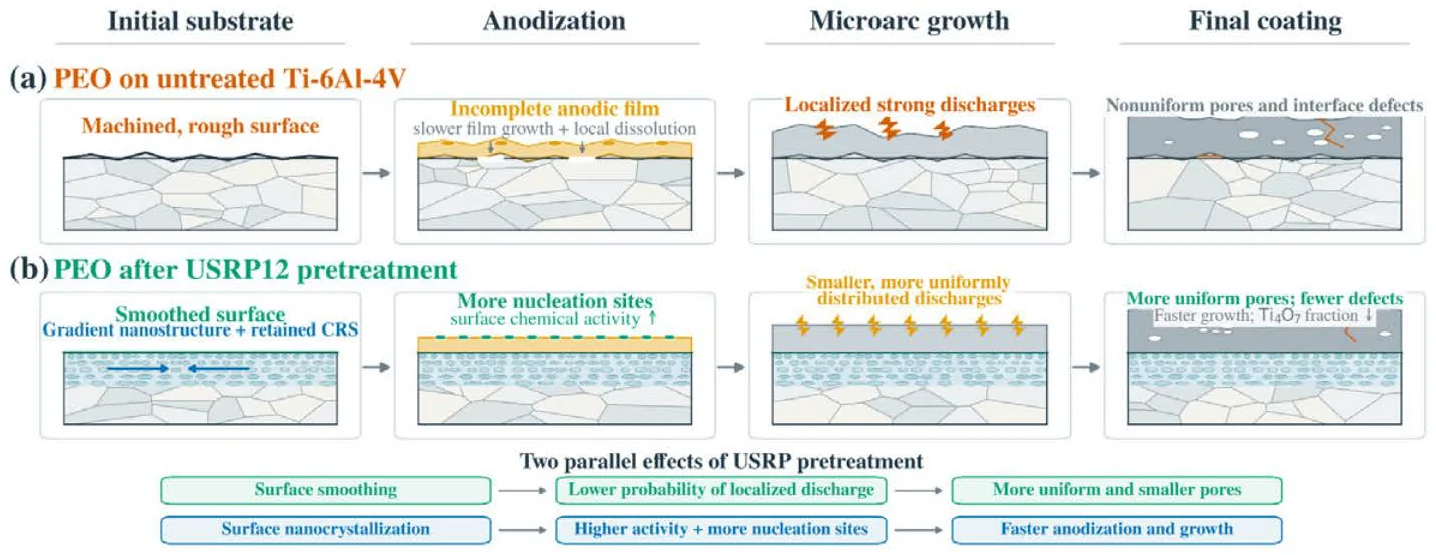
Schematic of PEO coating evolution on untreated and USRP12-pretreated Ti–6Al–4V: (**a**) relatively slow anodic film formation and localized strong discharges on the untreated rough substrate, leading to less uniform pores and more interfacial defects; and (**b**) two complementary effects of USRP12 pretreatment—surface smoothing reduces localized discharge and improves pore uniformity, whereas surface nanocrystallization increases surface activity and nucleation sites, accelerates anodization and coating growth, and lowers the Ti_4_O_7_ fraction. Both final coatings retain micropores and fine cracks. Developed by the authors based on mechanistic evidence reported in Ref. [59].

Pores, microcracks, and local tensile stresses in PEO coatings may serve as fatigue crack sources. USRP pretreatment reduces substrate roughness, produces a gradient nanostructure, and introduces compressive residual stress. As illustrated in Figure 11, pretreatment by 12 USRP passes (USRP12) affects PEO coating evolution through two complementary routes: surface smoothing reduces localized strong discharges and promotes a more uniform pore distribution, whereas surface nanocrystallization increases surface chemical activity and nucleation sites, thereby accelerating anodic film formation and coating growth [59]. PEO treatment was also reported to slightly relax the compressive residual stress near the outermost substrate surface while leaving the internal stress distribution largely unchanged [59]. For Ti–6Al–4V, combining USRP with an approximately 20 μm thick PEO coating produced a fatigue life at 600 MPa approximately 14 times that of the PEO-only specimen and an effective residual compressive stress layer approximately 180 μm deep [22]. A thicker PEO coating is, therefore, not necessarily better; coating thickness must be designed in coordination with compressive stress retention depth, coating defects, and interfacial stress state. Studies on related titanium alloys further reveal the role of this hybrid route in severe environments. For TC11 exposed at 500 °C with a NaCl deposit of 0.1 mg·cm^−2^, the hot salt environment reduced the substrate fatigue limit by 27.78%, whereas USRP, PEO, and USRP + PEO increased the hot salt corrosion fatigue limit by 10.26%, 47.44%, and 64.10%, respectively, relative to the substrate in the salt environment [60]. The dense PEO barrier layer inhibits chloride and hydrogen diffusion into the substrate, while the gradient microstructure and stable residual compressive stress introduced by USRP increase resistance to crack initiation and propagation.

Combining plasma zirconizing with USRP represents another interfacial control mechanism. Plasma zirconizing alone may reduce the high-temperature fatigue performance of TC11 because of surface microstructural weakening and stress concentration, whereas applying USRP after zirconizing reconditions the surface through refinement and residual compressive stress, increasing the fatigue limit at 500 °C by 7.3% relative to the substrate [61]. Pinning of dislocations by Zr solutes also improves the thermal stability of residual compressive stress. A clear process sequence effect, therefore, exists between coatings or diffusion layers and USRP, and surface chemistry, interfacial defects, substrate support, and residual stress stability must all be considered.

**Figure 12:**
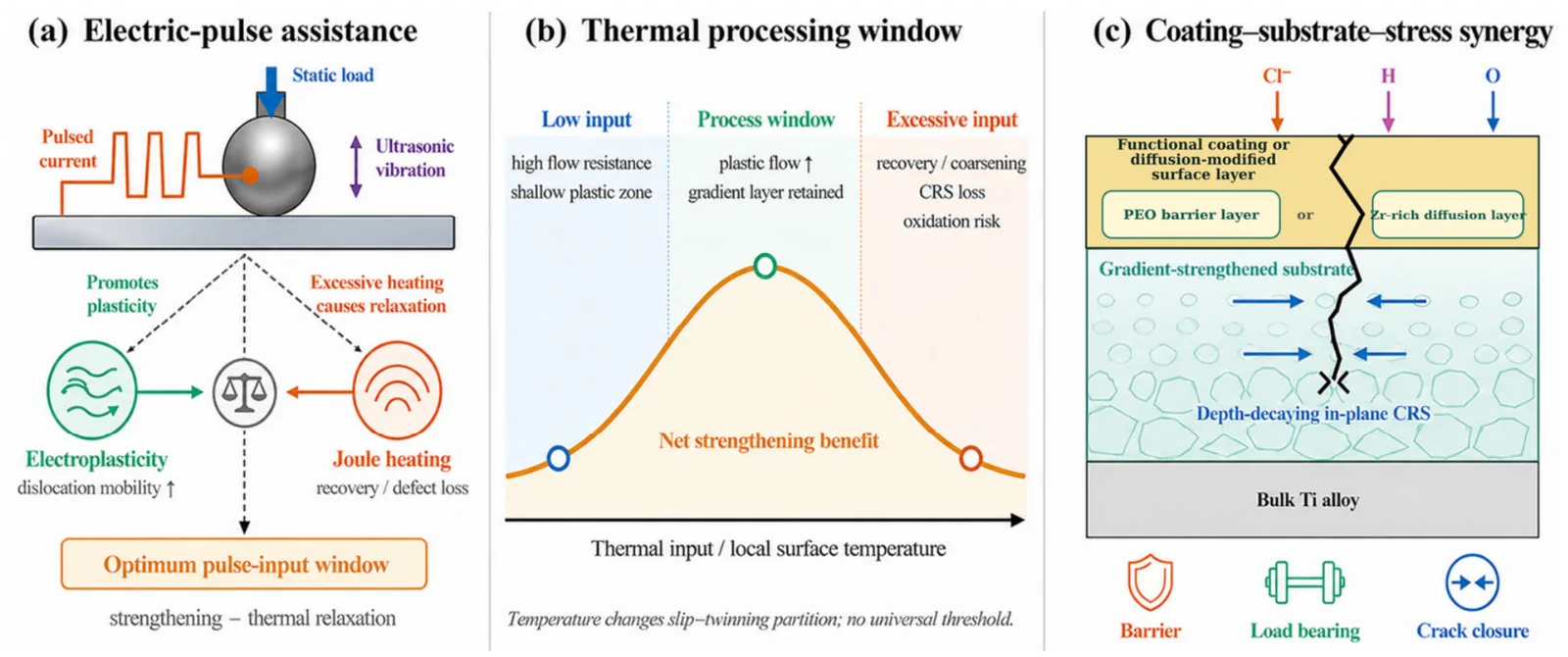
Competitive–synergistic mechanisms in hybrid USRP treatments: (**a**) competition between enhanced plastic deformation and Joule heating-induced recovery or stress relaxation under electropulsing assistance [23,52,53,54,55]; (**b**) the thermal-processing window between promoted plastic flow and excessive recovery, coarsening, residual stress loss, or oxidation [30,34,48,56,57]; and (**c**) interfacial synergy among a PEO barrier layer or Zr-rich diffusion layer, the gradient-strengthened substrate, and the residual compressive stress field [22,59,60,61]. The schematic was developed by the authors based on mechanistic evidence reported for Ti–6Al–4V and related titanium alloys.

As summarized in Figure 12, electropulsing-assisted, temperature-assisted, and coating or diffusion layer-coupled USRP act through different routes, but their effectiveness is governed by three common variables: the attainable plastic deformation depth, retention of the post-processing strengthened state, and stability of the interface and residual stress under service conditions. This understanding reconciles apparently contradictory outcomes such as “higher hardness but lower fatigue performance” and “a deeper deformation layer but lower residual compressive stress.” It also indicates that hybrid USRP should be assessed through multiple coordinated metrics—microstructural gradient, defect state, stress stability, and failure mode—rather than surface hardness alone.

To systematically compare the effects of conventional and hybrid USRP treatments on the surface integrity and fatigue performance of Ti–6Al–4V alloy, Table 1 summarizes representative results for surface roughness, residual compressive stress, hardened and deformation layer depths, microhardness, surface nanocrystallization, and fatigue life under different processing conditions.

Overall, USRP generally improves surface morphology, hardness gradient, and residual compressive stress simultaneously, but the strengthening effect does not increase monotonically with load, amplitude, or processing passes. Hybrid treatments can further broaden the microstructure and property control window; however, the initial material condition, process parameters, and fatigue-loading conditions differ considerably among studies. The tabulated results are, therefore, more suitable for comparison of trends than for simple numerical ranking.

## 7. Numerical Simulation and Data-Driven Optimization

USRP involves high-frequency impacts, rolling contact, nonlinear material plasticity, trajectory overlap, and unloading rebound, and some transient physical quantities are difficult to measure directly. Numerical simulation is valuable not only for reducing experimental cost but also for establishing quantitative links among process parameters, local loading, plastic strain, residual stress, and surface integrity. Data-driven methods can subsequently address multiparameter, strongly coupled, and multi-objective optimization problems.

### 7.1. Contact Dynamics and Residual Stress Prediction

Early studies primarily used three-dimensional explicit finite element models in which the rolling head was simplified as a rigid body and combined static pressure and ultrasonic vibration were represented by periodic displacement or loading, allowing prediction of equivalent plastic strain, hardened layer depth, and residual stress distribution [25]. Hertzian contact theory, elastoplastic constitutive models, and experimental calibration were subsequently introduced to improve model reliability [9,39,44].

Wang et al. established a three-dimensional finite element model of ultrasonic rolling of Ti–6Al–4V and separately analyzed the effects of static load, ultrasonic amplitude, and action time. Under their experimental and modeling conditions, a static load of 1100 N, an amplitude of 10 μm, and an action time of 0.065 s produced a relatively high surface residual compressive stress [39]. Li et al. found that residual compressive stress first increased and then decreased with rolling force, reaching a maximum near 600 N [9], whereas Ren et al. obtained favorable surface-smoothing parameters of 1000 N and 8 μm [44]. These different “optimal parameters” are not contradictory because they are jointly affected by rolling head radius, initial surface condition, constraints, material constitutive behavior, and the evaluation metric. Parameter combinations obtained by finite element analysis, therefore, cannot be generalized independently of the specific model.

The actual contact force during USRP is not a simple linear superposition of static and sinusoidal dynamic loads. Single-point cyclic indentation showed that the peak force during ultrasonic impact was more than 55% greater than the sum of the static and maximum dynamic loads. At indentation depths of 5, 10, and 15 μm, the nominal strain rates in the surface layer of Ti–6Al–4V were approximately 960, 1587, and 2043 s^−1^, respectively [8]. A quasi-static stress–strain curve may, therefore, underestimate the flow stress and hardening response under ultrasonic impact. A more reliable model should employ constitutive relations calibrated at different strain rates and validate the contact process against the dynamic force signal, indentation dimensions, and unloading rebound.

Trajectory overlap likewise determines the uniformity of the surface stress field. The model of Cong et al. showed that reducing the trajectory step from one-half to one-eighth of the indentation width decreased Ra by 39.68% and increased the plastic deformation layer depth to 56.07 μm. Further reduction to one-tenth produced a maximum residual compressive stress of −888.007 MPa but increased roughness [67]. Excessive coverage, therefore, causes repeated plastic deformation and surface material pile-up, and the parameter combination that maximizes residual compressive stress does not coincide with that which minimizes roughness.

Recent theoretical models have been extended to eccentric rolling trajectories. Tong et al. predicted residual stress on the TC4 surface using Hertzian contact and elastoplastic theory, with differences of 3.7–13.1% between theoretical and experimental values [31]. Current validation nevertheless remains focused mainly on surface residual stress or single-point hardness. Future studies should simultaneously validate dynamic contact force, three-dimensional surface morphology, the depth distribution of residual stress, and hardened layer thickness to avoid judging model validity from a single surface metric.

### 7.2. From Surface Deformation Prediction to Multiscale Microstructure Simulation

Most existing USRP models for Ti–6Al–4V assume a homogeneous isotropic continuum and approximate the deformation or hardened layer using an equivalent plastic strain region. Such models predict macroscopic stress and plastic zone dimensions but cannot directly describe α/β deformation differences, dislocation multiplication and annihilation, low-angle boundary formation, grain refinement, or the local hcp → fcc transformation. Converting equivalent plastic strain directly into grain size or hardness also requires independent experimental calibration and cannot be treated as a direct model output.

A more rational multiscale route should comprise three levels. First, a contact dynamics model should provide local histories of load, strain, strain rate, and temperature rise. Second, a dislocation density model, crystal plasticity finite element model, or microstructure evolution model should describe slip, twinning, dislocation storage, and grain refinement in the α and β phases. Finally, the microstructural gradient, hardness gradient, and residual stress field should be supplied to fatigue or wear models to predict crack initiation location and service life. Electropulsing-, laser-, and induction heating-assisted USRP additionally require temperature-dependent constitutive relations and models for thermal expansion, dynamic recovery, and thermal relaxation of residual stress.

Particular attention should be paid to the fact that fatigue performance after USRP is not determined solely by the initial surface residual compressive stress. Cyclic loading redistributes and relaxes residual stress, while excessive hardening may reduce plastic compatibility of the surface layer. Fatigue models should, therefore, include surface roughness and defect parameters together with depth-dependent hardness, grain size, and residual stress fields, rather than using only the maximum surface residual compressive stress as a life parameter.

Zhang et al. used surface roughness, residual stress gradient, hardness gradient, and fatigue-loading parameters as inputs to a preprocessing neural network for predicting the fatigue life of Ti–6Al–4V under different surface-strengthening conditions [35]. Internal feature dimensionality reduction retained depth-dependent information, and the model outperformed conventional machine learning models using principal component analysis. A random forest was also used to evaluate the contributions of individual surface integrity features. However, the dataset included different surface-strengthening methods, and the resulting feature importance represents statistical association rather than a substitute for physical analysis of residual stress relaxation and crack initiation.

### 7.3. Data-Driven Multi-Objective Optimization

USRP inputs include static load, amplitude, frequency, spindle speed, feed rate, processing passes, trajectory coverage, and temperature, while outputs include roughness, hardness, residual stress magnitude and depth, deformation layer thickness, and fatigue life. The input–output relationships are strongly nonlinear, and different performance objectives often compete; single-factor experiments and conventional orthogonal designs, therefore, have difficulty identifying a global optimum.

Combining finite element analysis with machine learning enables numerical simulations to augment the dataset. Pradhan et al. trained a Bayesian regularization model using finite element results for different materials and processing conditions and obtained coefficients of determination R^2^ of 0.973 and 0.926 for compressive and tensile stress states, respectively [68]. A surrogate trained on finite element data can, however, only approximate its parent finite element model. If the constitutive relation, contact boundary, or residual stress unloading procedure contains systematic bias, the machine learning model inherits the same error. Simulation data must, therefore, be jointly trained or corrected using independent experimental data. Chen et al. combined five machine learning models with NSGA-II and established a multi-objective ultrasonic-rolling parameter optimization framework that maximized residual compressive stress and hardness while minimizing roughness [69]. Although that study did not address Ti–6Al–4V, it provides a methodological basis for physics-informed feature enhancement and Pareto optimization under small-sample conditions.

A recent study on TC4 further used an improved whale optimization algorithm to tune RBF neural network parameters and combined a multi-objective gray wolf algorithm with CRITIC–TOPSIS for parameter decision-making [36]. After optimization, Ra, hardness, and residual stress reached 0.241 μm, 425.3 HV, and −868.4 MPa, respectively. Relative to the better combination in the orthogonal experiment, Ra decreased by 30.14%, hardness increased by 6.67%, and residual compressive stress increased by 16.52%; the maximum relative error between predictions and experiments was below 1.43%. These results show that data-driven methods can coordinate multiple surface property metrics. Nevertheless, the outputs were still limited to surface roughness, hardness, and surface residual stress and did not include strengthened layer depth, cyclic stability of residual stress, or fatigue life.

The next step in data-driven USRP research should, therefore, not be merely to adopt more complex optimization algorithms but to establish a closed-loop framework with physical constraints and uncertainty assessment. A standardized experimental database should provide the foundation, finite element analysis should generate supplemental samples, physics-informed machine learning should predict complete depth gradients, and a Pareto front should identify candidate parameters. A small number of validation experiments and online force–displacement signals should then update the framework. Such an approach could advance current experience-based offline parameter selection toward adaptive process control targeting residual stress stability and service life.

## 8. Engineering Applications, Technical Challenges, and Future Prospects

### 8.1. Application Scenarios Oriented Toward Service Failure Modes

USRP combines surface smoothing, gradient strengthening, and introduction of residual compressive stress and is, therefore, suitable for Ti–6Al–4V components in which fatigue cracks initiate mainly at or near the surface. Existing studies have addressed fretting contact regions, shafts and rolling contact components, welded joints, and additively manufactured components. However, the evidence remains dominated by standard and local feature specimens; it is, therefore, more accurate to state that USRP “has demonstrated engineering potential” than that it “has achieved large-scale engineering application.”

For fretting contact regions, such as blade roots and disk slots, the hardened layer and deep residual compressive stress formed by USRP reduce the effective tensile stress at contact edges and retard crack propagation [45,46,47]. Existing results, however, are derived mainly from flat or simplified contact specimens. Whether the rolling head can enter narrow slots reliably, whether edge regions undergo excessive deformation, and whether residual stress can be retained under complex contact loading must still be validated using actual structures.

Additively manufactured Ti–6Al–4V is a particularly promising application of USRP. Recent studies further show that the gradient microstructure produced by USRP not only improves surface integrity and rolling contact fatigue performance but also mitigates the strength–ductility trade-off by regulating strain partitioning between the surface and interior [70,71]. Studies of selective electron beam-melted and selective laser-melted materials show that USRP flattens adhered particles and melt pool ridges, forms a gradient-hardened layer, and improves rolling contact fatigue performance. Induction-heating assistance additionally increases the plastic-smoothing capability of rough as-built surfaces [20,27,29,30,42,71]. Nevertheless, build orientation, columnar prior β grains, internal pores, and lack-of-fusion defects may still govern final life, confirming that surface strengthening cannot replace control of internal metallurgical defects.

For Ti–6Al–4V laser-welded joints, USRP improves weld surface morphology, regulates welding residual stress, and reduces the fatigue crack growth rate, suppressing crack growth under both constant amplitude and overload conditions [21,72,73]. A similar overload retardation effect has been verified in unwelded TC4 specimens [74]. Existing experiments are still based mainly on welded coupons and precracked specimens, and systematic validation on actual welded components under complex load spectra and coupled high-temperature–corrosion environments is lacking. Laser-assisted UNSM has produced a marked fatigue life gain in additively repaired Ti–6Al–4V [75], but its contact mode and energy input differ from USRP, and it can only serve as evidence from an adjacent surface-strengthening technology for repaired components.

### 8.2. Key Bottlenecks from Specimen Strengthening to Component Application

First, stable normal contact and trajectory coverage are difficult to maintain on complex surfaces. Continuously changing curvature at blade leading edges, slots, weld toes, and thin-walled structures causes the actual contact force, indentation depth, impact density, and overlap ratio to vary along the path. Insufficient coverage may leave weakly strengthened regions, whereas excessive overlap may cause surface folding, hardening saturation, or residual stress relaxation. For complex components, the control variables should, therefore, shift from machine settings to the actual normal force, dynamic contact force, trajectory coverage, and energy input per unit area.

Second, the USRP process window depends strongly on the initial material condition and dominant failure mode. Forged, hot isostatically pressed, additively manufactured, and welded Ti–6Al–4V differ markedly in grain scale, phase constitution, texture, surface defects, and initial residual stress, and a single parameter set cannot be transferred directly among them. Process evaluation should not seek only the highest hardness, finest grains, or maximum residual compressive stress, but it should determine whether the strengthened layer depth covers the dominant damage zone and whether the gradient layer has sufficient deformation compatibility with the substrate.

Third, the stability of residual compressive stress during service remains insufficiently understood. Cyclic loading and fretting slip can redistribute or relax residual stress [14,45]. Thermal exposure and corrosive media can alter the residual stress state through microstructural recovery, oxidation, and surface damage, although a deep stress field and stable dislocation structure may improve thermo-mechanical retention; the actual evolution depends on processing passes, stress layer depth, and service environment [5,51]. Engineering assessment should, therefore, report not only the initial stress magnitude but also peak position, effective depth, directionality, and retention after service. For high-temperature or corrosive environments, the respective contributions of residual stress relaxation, microstructural recovery, and surface film breakdown to life should also be distinguished.

Fourth, after strengthening, the crack source may shift from the surface to the subsurface, where internal pores, coarse phase regions, or the gradient layer interface may become the new life-controlling locations. This failure transfer demonstrates that improved surface integrity does not necessarily imply improved damage tolerance of the entire component and requires nondestructive inspection to extend from surface defects to critical regions below the strengthened layer.

Finally, existing studies generally lack the statistical evidence required for engineering certification. Definitions of static load, amplitude measurement, rolling head dimensions, surface pretreatment, fatigue run-out cycles, and residual stress measurement methods are not standardized across the literature, while specimen number, batch repeatability, and life scatter are inadequately reported. Current results primarily demonstrate the feasibility of USRP and remain insufficient for establishing component design allowables or engineering safety factors directly.

### 8.3. Development Path Toward Engineering Certification

Future USRP research should shift from obtaining the highest hardness or maximum residual compressive stress toward designing surface integrity for the relevant service failure mode. Conventional fatigue requires priority to be given to reducing surface notch sensitivity and maintaining stable residual compressive stress. Fretting fatigue requires coordinated control of contact zone hardness, frictional state, and tangential stress. For rolling contact fatigue, the strengthened layer depth should cover the region of maximum cyclic shear stress. Corrosion fatigue requires simultaneous control of surface defects, gradient microstructure, residual stress, and passive film stability. These requirements define a reverse-design route linking service loading, dominant damage, target surface integrity, and process parameters.

At the equipment and modeling levels, multi-axis or robotic USRP systems suitable for complex surfaces should be developed, incorporating rolling head attitude, normal force, dynamic contact signals, surface temperature, and trajectory coverage into monitoring. Numerical models and data-driven methods should predict not only hardness or surface residual stress but also complete depth gradients, residual stress stability, crack initiation location, and outcome uncertainty, and they should be continuously corrected using independent experiments.

Engineering validation should follow a graded route from standard specimens to feature specimens, subcomponents, and full-scale components. Standard specimens should first establish a stable process window. Feature specimens containing notches, contact edges, welds, or additive manufacturing defects should then identify shifts in failure location. Tests should subsequently be conducted on blade roots, journals, and welded subcomponents, followed by full-scale validation under realistic temperature, corrosive media, and load spectra. Only when surface integrity, dimensional accuracy, residual stress stability, fatigue scatter, and inspection traceability all meet the requirements can USRP develop from a laboratory surface-strengthening method into a predictable, monitorable, and certifiable engineering-manufacturing technology.

## 9. Conclusions

This review systematically examined research advances, mechanisms, hybrid processes, and engineering issues associated with USRP of Ti–6Al–4V alloy within a “process parameters–gradient microstructure–surface integrity–service performance” framework. The main conclusions are as follows.

(1) Through the coupled action of static rolling force and high-frequency impacts, USRP introduces high-strain-rate plastic deformation into the surface layer of Ti–6Al–4V and forms a gradient structure consisting of nanocrystalline/ultrafine-grained layers, a plastically deformed layer, and the substrate. Microstructural refinement is governed mainly by dislocation multiplication, entanglement and rearrangement, subgrain boundary formation, and dynamic recovery, and the α and β phases follow different refinement paths. Local hcp → fcc transformation occurs only under specific stress states, crystallographic orientations, and deformation conditions and should not be regarded as a universal feature of USRP-treated material.
(2) The regulation of surface integrity by USRP exhibits pronounced parameter coupling and non-monotonic behavior. Appropriate static load, amplitude, feed, rotational speed, processing passes, and coverage can balance surface smoothing, microstructural refinement, work hardening, and introduction of residual compressive stress. Insufficient energy input cannot form an effective strengthened layer, whereas overprocessing may produce surface folding, delamination, local softening, and residual stress relaxation. Hardness, grain size, or peak residual compressive stress, therefore, cannot serve alone as process optimization criteria. Surface roughness, defect state, hardened layer depth, and the magnitude, distribution, and stability of residual stress should be assessed comprehensively.
(3) Fatigue improvement is currently the best-supported outcome of USRP research on Ti–6Al–4V and results from the combined effects of fewer surface defects, gradient-microstructure strengthening, and residual compressive stress. Residual compressive stress generally plays the dominant role by reducing effective tensile stress, increasing crack closure, and retarding early propagation, whereas gradient microstructure and work hardening mainly suppress surface plastic localization and crack initiation. After the crack source shifts into the subsurface, however, internal defects and the gradient layer interface may become new life-controlling factors. The effects of USRP on wear, erosion, and corrosion likewise depend on surface hardening, defect state, passive film stability, and the specific service environment, and do not necessarily increase monotonically with deformation severity.
(4) Hybrid processes involving electropulsing, auxiliary heating, laser assistance, deep cryogenic treatment, polishing, and PEO broaden the control range of conventional USRP, but their advantages arise from synergy among different physical fields rather than simple superposition of strengthening effects. Thermal or electrical assistance can reduce deformation resistance and deepen the strengthened layer, but it may also cause recovery, grain growth, oxidation, and attenuation of residual compressive stress. Hybrid processes should, therefore, define an appropriate energy-input window for the target failure mode and emphasize the long-term stability of the resulting microstructure and residual stress under thermal, mechanical, and corrosive environments.
(5) Existing finite element models can describe rolling contact, plastic deformation, and residual stress distributions, and data-driven methods have begun to be used for fatigue life prediction and multi-objective parameter optimization. However, a truly integrated multiscale model linking microstructural evolution, interphase deformation compatibility, residual stress relaxation, and cracking is still lacking. Future work should combine contact dynamics, crystal plasticity, microstructure evolution, and fracture models and introduce physics-constrained data-driven methods, predictive uncertainty, and online monitoring data to achieve closed-loop control of USRP on complex surfaces.

Overall, research on USRP of Ti–6Al–4V should move from pursuing “maximum hardness or the highest residual compressive stress” toward surface integrity design tailored to service failure modes. Unified parameter and characterization standards should be established, and validation under realistic load spectra, thermal exposure, corrosive environments, and batch repeatability conditions should proceed along the route of “standard specimen–feature specimen–subcomponent–full-scale component.” This progression will promote the development of USRP from a laboratory surface-strengthening method into a predictable, monitorable, and certifiable engineering-manufacturing technology.

## Figures and Tables

**Table 1:** Surface integrity and fatigue response of Ti–6Al–4V alloy under different USRP and hybrid treatment conditions.

Process and Specimen Condition	Ra (μm)	Maximum CRS (MPa) and Location	CRS-Affected Depth (μm)	Maximum Microhardness	Hardened Layer Depth (μm)	Microstructural/Deformation Layer Type and Depth (μm)	Nanocrystallization	Fatigue Response	Reference
USRP (16 passes)	0.231	−1001	—	462.6 HV	~400	plastic deformation layer 304.38	Not reported	—	[6]
USRP (specimen for hot salt corrosion study)	0.065 (before corrosion)	−948.6	~672	—	—	surface-refined layer~5	Yes	—	[5]
USRP	0.5	−1118; (500 μm)	—	580 ± 9 HK	400	140	Yes	Improved fretting fatigue performance; approximately 5.3-fold at 700 MPa	[47]
USRP (single pass)	0.108	−930; (50 μm)	~530	450 HK	~110	microstructurally refined layer~100–110	Not confirmed	Fretting fatigue life increased by 12.9-fold at 500 MPa relative to the untreated condition	[45]
USRP	0.143	~−550 (180 μm)	—	~374	—	—	Not reported	—	[4]
USRP	0.049	−954	~680	411 HV	150–200	plastic deformation layer~200	Yes	Approximately 8-fold at 700 MPa relative to the untreated condition (estimated from the original curve)	[17]
USRP	0.151	−960.4	~740	471.3 HV	~450	severely deformed layer~14.76	Not reported	Marked improvement at a stress level of 600 MPa	[38]
USRP (1 pass)	~0.102	surface: −963 MPa	approx. 540	approx. 425 HK	—	—	—	Fatigue limit (10^7^ cycles): 695 MPa (39% higher than BM); fatigue life at 700 MPa approximately 295 times that of BM	[3]
USRP (3 passes)	~0.123	surface: −956 MPa	~570	~450 HK	—	—	—	Fatigue limit (10^7^ cycles): 680 MPa (36% higher than BM)	[3]
USRP (6 passes)	~0.135	surface: −940 MPa	~595	~470 HK	—	—	—	Fatigue limit (10^7^ cycles): 670 MPa (34% higher than BM)	[3]
USRP (12 passes)	0.163	surface: −908 MPa	~620	509 HK	—	—	Yes	Fatigue limit (10^7^ cycles): 640 MPa (28% higher than BM); fatigue life at 700 MPa approximately 81.7 times that of BM	[3]
USRP	—	—	—	413 HV	~700	plastic deformation layer~300	Yes	—	[50]
USRP (12 passes)	0.19~0.24	surface: −259 MPa	—	412 HV	—	—	Yes	—	[62]
USRP	0.347	−867.7	—	508 HV	~500	gradient nanostructured layer~335	Yes	Fretting fatigue life increased by approximately 34.7-fold at 550 MPa relative to the untreated condition (estimated from the S–N curve)	[46]
URSP (near-net-shaped blade)	~0.20	subsurface peak: ~−800 (~30 μm)	~70	—	—	—	Not reported	—	[24]
USRP (1 pass, low deformation severity)	~0.62	surface: −1056 MPa	~600	450 HK_0.245_N	~100	—	No	Fatigue limit (10^7^ cycles): ~780 MPa; life at 850 MPa: ~167,333 cycles (approximately 19 times that of BM)	[16]
USRP (12 passes, high deformation severity)	~0.62	surface: ~−1000 MPa	~700	542 HK_0.245_N	~500	—	Yes	Fatigue limit (10^7^ cycles): ~740 MPa; life at 850 MPa: ~59,490 cycles (only approximately 4.8 times that of BM)	[16]
USRP (welded joint, 5 passes)	0.35	~−539	—	477 HV	—	deformation layer~31.93	Not reported	Approximately 1.67-fold relative to the untreated welded joint	[21]
USRP (120 °C)	0.2	~−491	—	414 HV	~60	—	Not reported	—	[56]
USRP (heat-treated HIP condition)	0.170 ± 0.025	−724 ± 23	—	467 ± 7.4 HV	~53	deformation layer~20	did not reach the nanoscale	Several-fold at 700 MPa relative to the untreated condition	[63]
USRP (SLM condition, 1000 N)	—	—	—	—	—	plastic deformation layer~300	Yes	—	[27]
USRP	—	—	—	—	—	gradient microstructural layer~400; fcc-Ti detectable to~440	Yes	—	[13]
USRP	—	—	—	~580 HK	—	gradient plastic deformation layer >400	Yes	—	[11]
USRP (fretting fatigue specimen)	0.358	~−1099 (60 μm)	~700	563 HK	~120	gradient deformation layer~160	Not reported	Approximately 5.2-fold at 650 MPa relative to the untreated condition (estimated from the S–N curve)	[15]
USRP (SLM condition, 1000 N, 6 passes)	—	—	—	~422 HV	~200	densified/pore closure layer~100–200	Not reported	—	[42]
MUSR	0.13	~−1173	—	435 HV	~55	strongly affected depth~450; surface severely deformed zone~35	Not reported	From approximately twofold to approximately two orders of magnitude at 650–560 MPa relative to the untreated condition	[64]
MUSR (alternative frequency/load combination)	0.15	~−1110	—	455 HV	~38	surface severely deformed zone~30	Not reported	—	[26]
USRP	0.718	~−1155 (125 μm)	~650	521 HK	~500	gradient plastic deformation layer~335; surface equiaxed nanograined layer ≥10	Yes	23.5-fold at 700 MPa relative to the untreated condition	[14]
USRP + polishing	—	~−937.6 (surface)	—	524 HK	—	gradient nanostructure retained	Yes	113.8-fold at 700 MPa relative to the untreated condition	[14]
USRP-MM	—	—	—	—	—	—	Not reported	Approximately fourfold at 700 MPa relative to the damaged condition	[65]
USRP (pretreatment before PEO)	0.049	~−941	>560	—	~150	nanocrystalline surface layer	Yes	—	[22]
USRP–PEO (20 μm coating)	2.553 (coating surface)	~−590	~180	~780 HV (coating)	—	interfacial grain size~81 nm	Yes	Approximately 14-fold at 600 MPa relative to PEO alone	[22]
USRP–PEO	1.5 ± 0.2 (coating surface)	−1167 ± 58 (50 μm); interface−835 ± 34	—	—	—	nanocrystalline structure retained at the interface	Yes	Approximately 2.3-fold at 800 MPa	[59]
DC-USRP (SLM condition)	~0.18	~−740 (surface)	—	—	—	—	Not reported	—	[54]
EUSRP-2 + USRP (200 A)	0.174	~−716	—	417 HV	~600	gradient deformation layer~385–400	Yes	Approximately 25-fold at 780 MPa relative to the untreated condition	[23]
EP-USRP	~0.20	axial ~ −393; circumferential ~−554	—	468 HV	~420	plastic deformation layer >100	Not confirmed	—	[52]
HUR (140 °C)	~0.30	—	—	495 HV	~260	deformation layer~30	Not reported	—	[57]
HUSS (heating-assisted)	~0.23	~−955 (140 μm)	—	513 HV	~180	nanocrystalline layer~50	Yes	—	[66]
IH-USRP (650 °C)	~0.70	−1107 ± 154	—	406.08 HV	—	deformation layer~59.8	Not reported	Rolling contact fatigue life increased by 2.96-fold relative to the untreated condition	[30]
DCT + USRP (−196 °C × 24 h)	0.099	~−460	—	nanohardness 5.78 GPa	—	severely deformed/nanostructured layer~7.8	Yes	—	[58]

CRS denotes residual compressive stress; “—“ indicates that the original article did not report the value or that the available evidence was insufficient to attribute it to the listed treatment condition.

## Data Availability

No new data were created or analyzed in this study. Data sharing is not applicable to this article.

## References

[1] Khanna N., Davim J.P. (2015). Design-of-experiments application in machining titanium alloys for aerospace structural components. Measurement.

[2] Xie B., Gao K. (2023). Research Progress of Surface Treatment Technologies on Titanium Alloys: A Mini Review. Coatings.

[3] Liu C., Liu D., Zhang X., He G., Xu X., Ao N., Ma A., Liu D. (2019). On the influence of ultrasonic surface rolling process on surface integrity and fatigue performance of Ti-6Al-4V alloy. Surf. Coat. Technol..

[4] Wang N., Zhu J., Liu B., Zhang X., Zhang J., Tu S. (2021). Influence of Ultrasonic Surface Rolling Process and Shot Peening on Fretting Fatigue Performance of Ti-6Al-4V. Chin. J. Mech. Eng..

[5] Li M., Liu D., Bai Z., Fan K., Yang J., Zhang X., Shi H. (2023). Hot salt corrosion behavior of Ti–6Al–4V alloy treated with different surface deformation strengthening processes. Mater. Chem. Phys..

[6] Luo X., Ren X., Jin Q., Qu H., Hou H. (2021). Microstructural evolution and surface integrity of ultrasonic surface rolling in Ti6Al4V alloy. J. Mater. Res. Technol..

[7] Zhu X., Liu P., Zhang C., Liang H., Hua J. (2023). Study on Surface Integrity and Fatigue Properties of TC4 Titanium Alloy by Surface Ultrasonic Rolling. Materials.

[8] Zha X., Yuan Z., Qin H., Xi L., Guo B., Zhang T., Jiang F. (2024). Coupling mechanisms of static and dynamic loads during the ultrasonic impact strengthening of Ti-6Al-4V. Int. J. Adv. Manuf. Technol..

[9] Li F., Zhao B., Lan S., Feng Z. (2020). Experiment and simulation of the effect of ultrasonic rolling on the surface properties of Ti-6Al-4V. Int. J. Adv. Manuf. Technol..

[10] Lu K. (2014). Making strong nanomaterials ductile with gradients. Science.

[11] Ao N., Liu D., Xu X., Zhang X., Liu D. (2019). Gradient nanostructure evolution and phase transformation of α phase in Ti-6Al-4V alloy induced by ultrasonic surface rolling process. Mater. Sci. Eng. A.

[12] Ao N., Liu D., Zhang X., Liu C., Yang J., Liu D. (2019). Surface nanocrystallization of body-centered cubic beta phase in Ti–6Al–4V alloy subjected to ultrasonic surface rolling process. Surf. Coat. Technol..

[13] Ao N., Liu D., Liu C., Zhang X., Liu D. (2018). Face-centered titanium induced by ultrasonic surface rolling process in Ti-6Al-4V alloy and its tensile behavior. Mater. Charact..

[14] Liu C., Liu D., Zhang X., Liu D., Ma A., Ao N., Xu X. (2019). Improving fatigue performance of Ti-6Al-4V alloy via ultrasonic surface rolling process. J. Mater. Sci. Technol..

[15] Fan K., Liu D., Zhang X., Liu D., Zhao W., Yang J., Ma A., Li M., Qi Y., Xiang J. (2022). Effect of residual stress induced by ultrasonic surface rolling on fretting fatigue behaviors of Ti-6Al-4V alloy. Eng. Fract. Mech..

[16] Ao N., Liu D., Zhang X., Zhang J., Wu S. (2022). Surface rolling deformed severity-dependent fatigue mechanism of Ti-6Al-4V alloy. Int. J. Fatigue.

[17] Pang Z., Wang S., Yin X., Yu S., Du N. (2022). Effect of spindle speed during ultrasonic rolling on surface integrity and fatigue performance of Ti6Al4V alloy. Int. J. Fatigue.

[18] Wang S., Yu T., Pang Z., Yin X., Liu X. (2023). Effect of Repeated Processing Passes during Ultrasonic Rolling on Fatigue Performance and Corrosion Resistance of Ti6Al4V Alloy. Metals.

[19] Zha X., Qin H., Yuan Z., Xi L., Chen X., Li Y., Jiang Q., Xu Z., Jiang F. (2025). Surface integrity and fatigue properties of Ti–6Al–4V alloy under the ultrasonic surface rolling process excited strain rate effect. J. Mater. Res. Technol..

[20] Liu Z., Gao C., Liu X., Liu R., Xiao Z. (2021). Improved surface integrity of Ti6Al4V fabricated by selective electron beam melting using ultrasonic surface rolling processing. J. Mater. Process. Technol..

[21] Cong J., Gao J., Zhou S., Wang N., Wang J., Li H. (2023). Effect of ultrasonic rolling on crack propagation behavior of Ti6Al4V titanium alloy laser welded joints. Eng. Fract. Mech..

[22] Wang S., Yu T., Pang Z., Liu X., Shi C., Du N. (2024). Improving the fatigue resistance of plasma electrolytic oxidation coated titanium alloy by ultrasonic surface rolling pretreatment. Int. J. Fatigue.

[23] Sun P., Qu S., Duan C., Hu X., Li X. (2024). Improving the high cycle fatigue property of Ti6Al4V ELI alloy by optimizing the surface integrity through electric pulse combined with ultrasonic surface rolling process. J. Mater. Sci. Technol..

[24] Wu D., Lv H., Wang H., Yu J. (2022). Surface micro-morphology and residual stress formation mechanisms of near-net-shaped blade produced by low-plasticity ultrasonic rolling strengthening process. Mater. Des..

[25] Liu Y., Wang L., Wang D. (2011). Finite element modeling of ultrasonic surface rolling process. J. Mater. Process. Technol..

[26] Li G., Qu S.G., Pan Y.X., Li X.Q. (2016). Effects of the different frequencies and loads of ultrasonic surface rolling on surface mechanical properties and fretting wear resistance of HIP Ti–6Al–4V alloy. Appl. Surf. Sci..

[27] Wang Z., Gao C., Liu Z., Wang Z., Liu X., Wong K., Zhou Z., Xiao Z. (2020). Investigation of microstructural evolution in a selective laser melted Ti6Al4V alloy induced by an ultrasonic surface rolling process. Mater. Sci. Eng. A.

[28] Sun P., Qu C., Zhong H., Duan C., Li X., Qu S. (2024). Quantitative mechanism of abnormal hardening behavior of Ti6Al4V alloy strengthened by ultrasonic surface rolling. Surf. Coat. Technol..

[29] Liu Z., Wang Z., Gao C., Liu X., Liu R., Xiao Z., Sanderson J. (2022). Enhanced rolling contact fatigue behavior of selective electron beam melted Ti6Al4V using the ultrasonic surface rolling process. Mater. Sci. Eng. A.

[30] Liu Z., Liu X., Liu R., Xiao Z., Sanderson J. (2023). Improved rolling contact fatigue performance of selective electron beam melted Ti6Al4V with the as-built surface using induction-heating assisted ultrasonic surface rolling process. Appl. Surf. Sci..

[31] Tong J., Tao H., Yang S., Ye Y., Zhai H., Li X., Liu L., Zheng Y., Song C. (2025). Modeling and Experimental Validation of Residual Stresses on the Surface of TC4 Titanium Alloy by Eccentric Ultrasonic Surface Rolling Process. Int. J. Precis. Eng. Manuf..

[32] Tong J., Zheng Y., Yang S., Li X., Liu L., Xiang D., Gao G. (2026). Research on the Surface Characteristics of TC4 Titanium Alloy Subjected to Eccentric Ultrasonic Surface Rolling Process. J. Mater. Eng. Perform..

[33] Zha X., Chen X., Qin H., Li J., Guo Y., Zhang T., Jiang F. (2026). Effect of ultrasonic rolling coverage rate on surface integrity and strengthening mechanisms of Ti–6Al–4V alloy by the trajectory interference experiments. J. Mater. Res. Technol..

[34] Li Y., Zhang H., Liu D., Zhang Y., Jia T., Ye C., Zhang X. (2025). Laser-assisted ultrasonic surface rolling of titanium alloys: Governing the softening-strengthening transition for fatigue enhancement. J. Alloys Compd..

[35] Zhang Y., Wang X.-K., Jia Y.-F., Dong B., Wang Z.-M., Yan J.-J., Zhang X.-C., Tu S.-T. (2025). Fatigue Life Prediction and Feature Contribution Analysis of Surface-Strengthened Ti–6Al–4V Using a Preprocessing Neural Network. Metall. Mater. Trans. A.

[36] Lan Y., Rao C., Lyu Y. (2026). Multi-Objective Optimization of Ultrasonic Surface Rolling Process Parameters for TC4 Titanium Alloy with IWOA–RBF and MOGWO Algorithms. Micromachines.

[37] Huang J., Zhang Z., Yang J., Wen X., Liu D., Li T., Wan M., Chen J., Huang C. (2026). Ultrasonic surface rolling improves tensile properties but reduces rotating bending fatigue strength in a titanium alloy. J. Alloys Compd..

[38] Zhang K.-M., Liu S., Wang J., Sun Z.-X., Liu W.-J., Zhang C.-C., Zhang X.-C. (2024). Effect of high-frequency dynamic characteristics in the ultrasonic surface rolling process on the surface properties. J. Mater. Process. Technol..

[39] Wang F., Men X., Liu Y., Fu X. (2020). Experiment and simulation study on influence of ultrasonic rolling parameters on residual stress of Ti-6Al-4V alloy. Simul. Model. Pract. Theory.

[40] Zha X., Yuan Z., Qin H., Xi L., Guo Y., Xu Z., Dai X., Jiang F. (2024). Investigating the Dynamic Mechanical Properties and Strengthening Mechanisms of Ti-6Al-4V Alloy by Using the Ultrasonic Surface Rolling Process. Materials.

[41] Wang Y., Liu Y., Wang D., Ma J., Han J. (2024). Research on surface integrity of TC4 titanium alloy by ultrasonic surface rolling peening. Int. J. Adv. Manuf. Technol..

[42] Wang Z., Xiao Z., Huang C., Wen L., Zhang W. (2017). Influence of Ultrasonic Surface Rolling on Microstructure and Wear Behavior of Selective Laser Melted Ti-6Al-4V Alloy. Materials.

[43] Wang E., Zhou F., Zhou L., Jiang Z., Xu P., Guo Y., Luo J., Tang L., Chen Y., Li X. (2025). The effect of partial surface strengthening on the strength and plasticity of Ti6Al4V titanium alloy. Mater. Sci. Eng. A.

[44] Ren Z., Li Z., Zhou S., Wang Y., Zhang L., Zhang Z. (2022). Study on surface properties of Ti-6Al-4V titanium alloy by ultrasonic rolling. Simul. Model. Pract. Theory.

[45] Liu C., Liu D., Zhang X., Yu S., Zhao W. (2017). Effect of the Ultrasonic Surface Rolling Process on the Fretting Fatigue Behavior of Ti-6Al-4V Alloy. Materials.

[46] Liu C., Liu D., Zhang X., Ao N., Xu X., Liu D., Yang J. (2019). Fretting fatigue characteristics of Ti-6Al-4V alloy with a gradient nanostructured surface layer induced by ultrasonic surface rolling process. Int. J. Fatigue.

[47] Ao N., Liu D., Zhang X., Wu S. (2023). Improved fretting fatigue mechanism of surface-strengthened Ti-6Al-4V alloy induced by ultrasonic surface rolling process. Int. J. Fatigue.

[48] Fan K., Liu D., Zhou K., Liu Y., Zhang H., Zhang X., Li Y., Li M., Li Y., Abdel Wahab M. (2024). Enhanced fretting fatigue strength of TC11 titanium alloy using laser-assisted ultrasonic surface rolling process. Tribol. Int..

[49] Zheng K., Zhao X., Pan L., Ren Z. (2024). Ultrasonic rolling strengthening of TC11 titanium alloy surface: Corrosion and wear properties under extreme conditions. Wear.

[50] Shi Y., Liu Y., Li X., Zhang Y. (2018). Effect of Ultrasonic Surface Rolling Process on Solid Particles Erosion Performance of Ti-6Al-4V. IOP Conf. Ser. Mater. Sci. Eng..

[51] Li M., Liu D., Zhou K., Liu Y., Yang Z., Wu J., Zhang X. (2025). Surface integrity engineering via ultrasonic surface rolling for enhanced hot salt stress corrosion cracking resistance of TC11 alloy. Surf. Coat. Technol..

[52] Wang H., Song G., Tang G. (2016). Evolution of surface mechanical properties and microstructure of Ti 6Al 4V alloy induced by electropulsing-assisted ultrasonic surface rolling process. J. Alloys Compd..

[53] Qu S., Ren Z., Hu X., Lai F., Sun F., Li X., Yang C. (2021). The effect of electric pulse aided ultrasonic rolling processing on the microstructure evolution, surface properties, and fatigue properties of a titanium alloy Ti5Al4Mo6V2Nb1Fe. Surf. Coat. Technol..

[54] Wang Z., Liu Z., Gao C., Wong K., Ye S., Xiao Z. (2020). Modified wear behavior of selective laser melted Ti6Al4V alloy by direct current assisted ultrasonic surface rolling process. Surf. Coat. Technol..

[55] Li G., Hong B., Song D., Ma Y., Yuan C. (2025). Effect of pulsed current coupled ultrasonic rolling on surface fretting friction and wear properties of Ti-6Al-4V alloy. Wear.

[56] Li G., Qu S., Xie M., Li X. (2017). Effect of ultrasonic surface rolling at low temperatures on surface layer microstructure and properties of HIP Ti-6Al-4V alloy. Surf. Coat. Technol..

[57] Li G., Meng F., Zhang W. (2023). Effect of Heating-Assisted Ultrasonic Rolling on Surface Properties of Ti-6Al-4V Alloy. JOM.

[58] Luo X., Ren X., Qu H., Hou H., Chen J., Tian P. (2022). Research on influence of deep cryogenic treatment and ultrasonic rolling on improving surface integrity of Ti6Al4V alloy. Mater. Sci. Eng. A.

[59] Ao N., Liu D., Zhang X., Liu C. (2019). Enhanced fatigue performance of modified plasma electrolytic oxidation coated Ti-6Al-4V alloy: Effect of residual stress and gradient nanostructure. Appl. Surf. Sci..

[60] Shi H., Liu D., Jia T., Zhang X., Zhao W. (2023). Effect of the ultrasonic surface rolling process and plasma electrolytic oxidation on the hot salt corrosion fatigue behavior of TC11 alloy. Int. J. Fatigue.

[61] Wu J., Liu D., Zhang X., Liu Y., Yang Z., Xiang J. (2025). Enhancing high-temperature fatigue resistance of TC11 titanium alloy through combined plasma zirconizing and ultrasonic surface rolling. Int. J. Fatigue.

[62] Wang P., Pan Y., Liu Y., Fu X., Li H. (2021). Research on surface properties of Ti-6Al-4V alloy by multi-ultrasonic rolling. Proc. Inst. Mech. Eng. Part C J. Mech. Eng. Sci..

[63] Li G., Qu S., Guan S., Wang F. (2019). Study on the tensile and fatigue properties of the heat-treated HIP Ti-6Al-4V alloy after ultrasonic surface rolling treatment. Surf. Coat. Technol..

[64] Li G., Qu S., Xie M., Ren Z., Li X. (2017). Effect of Multi-Pass Ultrasonic Surface Rolling on the Mechanical and Fatigue Properties of HIP Ti-6Al-4V Alloy. Materials.

[65] Ao N., Liu D., Zhang X. (2020). Improving fatigue performance of surface-damaged Ti-6Al-4V alloy via ultrasonic surface rolling process. MATEC Web Conf..

[66] Li G., Xiao X., Zhang W., Song D. (2023). Study on surface fretting friction properties of heat-treated HIP Ti-6Al-4 V alloy after heating-assisted ultrasonic surface strengthening. J. Mater. Process. Technol..

[67] Cong J., Gao J., Wang J., Wang L., Li H. (2023). Analysis of the residual stress and surface morphology of TI6AL4V using elastoplastic contact ultrasonic rolling. Proc. Inst. Mech. Eng. Part C J. Mech. Eng. Sci..

[68] Pradhan R., Altalbawy F.M.A., Khan A.R., Rodriguez-Benites C., Sharma M.K., Asaad R.R. (2024). A FEM-guided data-driven machine learning model for residual stress characterization in ultrasonic surface rolling of lightweight alloys. Appl. Phys. A.

[69] Chen J., Yang T., Chen S., Jiang Q., Li Y., Chen X., Xu Z. (2024). Multi-Objective Process Parameter Optimization of Ultrasonic Rolling Combining Machine Learning and Non-Dominated Sorting Genetic Algorithm-II. Materials.

[70] Liu Z., Lu H., Cai C., Luo Y., Xu G., Mao W., Zhou Z., Xiao Z. (2025). Simultaneously improved strength and ductility of selective electron beam melted Ti6Al4V using ultrasonic surface rolling process. J. Mater. Res. Technol..

[71] Zhou C., Yan X., Liu D., Xu X., Cui J., Li M., Yuan C., Li H., Liang Y. (2025). Simultaneously achieving strength-ductility in additive-manufactured Ti6Al4V alloy via ultrasonic surface rolling process. Mater. Sci. Eng. A.

[72] Cong J., Gao J., Zhou S., Zhang Z., Wang J., Wang N. (2024). Effect of ultrasonic rolling on the fatigue performance of laser-welded TC4 titanium alloy joints. Int. J. Fract..

[73] Gao J., Cong J., Zhou S., Zhang Z., Gao S., Liu Z. (2025). Effect of ultrasonic rolling on crack growth behavior of TC4 titanium alloy laser weld under overload condition. Theor. Appl. Fract. Mech..

[74] Cong J., Gao S., Zhou S., Zhang Z., Zhu X., Liu Z. (2025). Influence of ultrasonic rolling on crack growth behavior of TC4 titanium alloy under various tensile overload conditions. Theor. Appl. Fract. Mech..

[75] Ojo S.A., Bowser B., Manigandan K., Morscher G.N., Dong Y., Gyekenyesi A.L., Scott-Emuakpor O.E. (2023). Improving fatigue life of additively repaired Ti-6Al-4V subjected to laser-assisted ultrasonic nanocrystal surface modification. Int. J. Fatigue.

